# Mycobacterium tuberculosis PE_PGRS20 and PE_PGRS47 Proteins Inhibit Autophagy by Interaction with Rab1A

**DOI:** 10.1128/mSphere.00549-21

**Published:** 2021-08-04

**Authors:** Emily J. Strong, Tony W. Ng, Steven A. Porcelli, Sunhee Lee

**Affiliations:** a Department of Microbiology and Immunology, University of Texas Medical Branch, Galveston, Texas, USA; b Department of Microbiology and Immunology, Albert Einstein College of Medicine, Bronx, New York, USA; c Department of Medicine, Albert Einstein College of Medicine, Bronx, New York, USA; d Department of Molecular Genetics and Microbiology, Duke University, Durham, North Carolina, USA; Washington University School of Medicine in St. Louis

**Keywords:** *Mycobacterium tuberculosis*, PE_PGRS proteins, autophagy, host-pathogen interactions, innate immunity

## Abstract

Autophagy is a fundamental cellular process that has important roles in innate and adaptive immunity against a broad range of microbes. Many pathogenic microbes have evolved mechanisms to evade or exploit autophagy. It has been previously demonstrated that induction of autophagy can suppress the intracellular survival of mycobacteria, and several PE_PGRS family proteins of Mycobacterium tuberculosis have been proposed to act as inhibitors of autophagy to promote mycobacterial survival. However, the mechanisms by which these effectors inhibit autophagy have not been defined. Here, we report detailed studies of M. tuberculosis deletion mutants of two genes, *pe_pgrs20* and *pe_pgrs47*, that we previously reported as having a role in preventing autophagy of infected host cells. These mutants resulted in increased autophagy and reduced intracellular survival of M. tuberculosis in macrophages. This phenotype was accompanied by increased cytokine production and antigen presentation by infected cells. We further demonstrated that autophagy inhibition by PE_PGRS20 and PE_PGRS47 resulted from canonical autophagy rather than autophagy flux inhibition. Using macrophages transfected to express PE_PGRS20 or PE_PGRS47, we showed that these proteins inhibited autophagy initiation directly by interacting with Ras-related protein Rab1A. Silencing of Rab1A in mammalian cells rescued the survival defects of the *pe_pgrs20* and *pe_pgrs47* deletion mutant strains and reduced cytokine secretion. To our knowledge, this is the first study to identify mycobacterial effectors that directly interact with host proteins responsible for autophagy initiation.

**IMPORTANCE** Tuberculosis is a significant global infectious disease caused by infection of the lungs with Mycobacterium tuberculosis, which then resides and replicates mainly within host phagocytic cells. Autophagy is a complex host cellular process that helps control intracellular infections and enhance innate and adaptive immune responses. During coevolution with humans, M. tuberculosis has acquired various mechanisms to inhibit host cellular processes, including autophagy. We identified two related M. tuberculosis proteins, PE_PGRS20 and PE_PGRS47, as the first reported examples of specific mycobacterial effectors interfering with the initiation stage of autophagy. Autophagy regulation by these PE_PGRS proteins leads to increased bacterial survival in phagocytic cells and increased autophagic degradation of mycobacterial antigens to stimulate adaptive immune responses. A better understanding of how M. tuberculosis regulates autophagy in host cells could facilitate the design of new and more effective therapeutics or vaccines against tuberculosis.

## INTRODUCTION

Mycobacterium tuberculosis is an extraordinarily successful host-adapted pathogen that causes tuberculosis (TB), a severe infectious disease with high morbidity and mortality. In 2019, 1.4 million deaths were directly attributed to M. tuberculosis infection, and a further 10 million people developed active TB (1, 2). M. tuberculosis has evolved many effective survival strategies as a primarily intracellular parasite of host phagocytic cells. One of its best-studied survival mechanisms is phagolysosome maturation and acidification (3). However, substantial evidence shows that other host defense mechanisms, including autophagy and apoptosis, are also inhibited by M. tuberculosis infection (4–6).

Autophagy is a ubiquitous and fundamental cellular process and is an important control mechanism against intracellular microbes. Autophagy can initiate phagosome maturation and enhance the processing and presentation of antigens to T cells, increasing bacterial clearance through innate and adaptive immunity. Regulation and modulation of autophagy are complex and affected by many feedback mechanisms, most of which are not fully elucidated. Stimulation of autophagy by starvation or by its critical regulator mechanistic target of rapamycin (mTOR) by drugs such as rapamycin results in colocalization of intracellular bacteria to autophagosomes and, consequently, increased bacterial clearance (4–10).

Bacterial clearance by autophagy is termed xenophagy and will be referred to as autophagy here. M. tuberculosis cytosolic DNA is recognized by the cytosolic DNA sensor, cyclic GMP-AMP synthase (cGAS), resulting in the release of cyclic GMP (cGMP). cGAMP is recognized by the stimulator of interferon genes (STING), leading to the recruitment of ubiquitin receptors p62, NDP52, and optineurin, along with type I interferon (IFN) release (11, 12). These receptors are recruited to the ubiquitinated pathogen, thereby allowing for specific targeting by the autophagosome (13–15). While autophagy is an effective mechanism for clearing bacteria, some bacterial pathogens possess effectors inhibiting its induction from promoting their survival. In mycobacteria, multiple such effectors have been identified, including the secreted protein EIS (enhanced intracellular survival) (16, 17) and several virulence-associated M. tuberculosis PE_PGRS and PPE family proteins (6, 18, 19). The mechanisms by which these mycobacterial effectors may inhibit autophagy are not well understood.

The PE/PPE family of mycobacterial proteins is found most abundantly in slow-growing pathogenic mycobacteria and constitutes approximately 10% of the M. tuberculosis genome (20). It has been previously demonstrated that the PE/PPE proteins of M. tuberculosis are partially responsible for inhibiting autophagy in phagocytic cells upon infection with mycobacteria (6, 18, 19, 21). This family of proteins has coevolved with the ESX type VII secretion systems present in mycobacteria, which are prominently involved in virulence and intracellular survival of mycobacteria (22–24). Several PE/PPE proteins have been implicated as autophagy inhibitors in M. tuberculosis-infected cells, thus contributing to the virulence and persistence of the bacteria (6, 18, 19, 21). Our previous screen of a random transposon mutant library of M. tuberculosis identified PE_PGRS20 and PE_PGRS47 as two such autophagy-inhibiting factors.

In the current study, we focused on determining how M. tuberculosis PE_PGRS20 and PE_PGRS47 proteins inhibit autophagy. We found that M. tuberculosis strains with genetic deletions of *pe_pgrs20* and *pe_pgrs47* induced increased autophagy and reduced bacterial survival in human monocyte-derived macrophages and murine bone marrow-derived macrophages. While confirming that this autophagy induction was reliant on canonical autophagy and the previously described role for PE_PGRS20 and PE_PGRS47 in mTOR modulation (18), the current study identified a functionally relevant direct interaction of these PE_PGRS proteins with Rab1A, a host protein required for autophagy initiation. In addition to blocking autophagy, the interaction of Rab1A with PE_PGRS20 or PE_PGRS47 reduced secretion of proinflammatory cytokines and inhibited major histocompatibility complex (MHC) class II-restricted antigen presentation. These results demonstrate related effects on innate and adaptive immunity that are likely to contribute to bacterial escape from host defenses during M. tuberculosis infection.

## RESULTS

### PE_PGRS20 and PE_PGRS47 of M. tuberculosis inhibit autophagy in macrophages.

**FIG 1:**
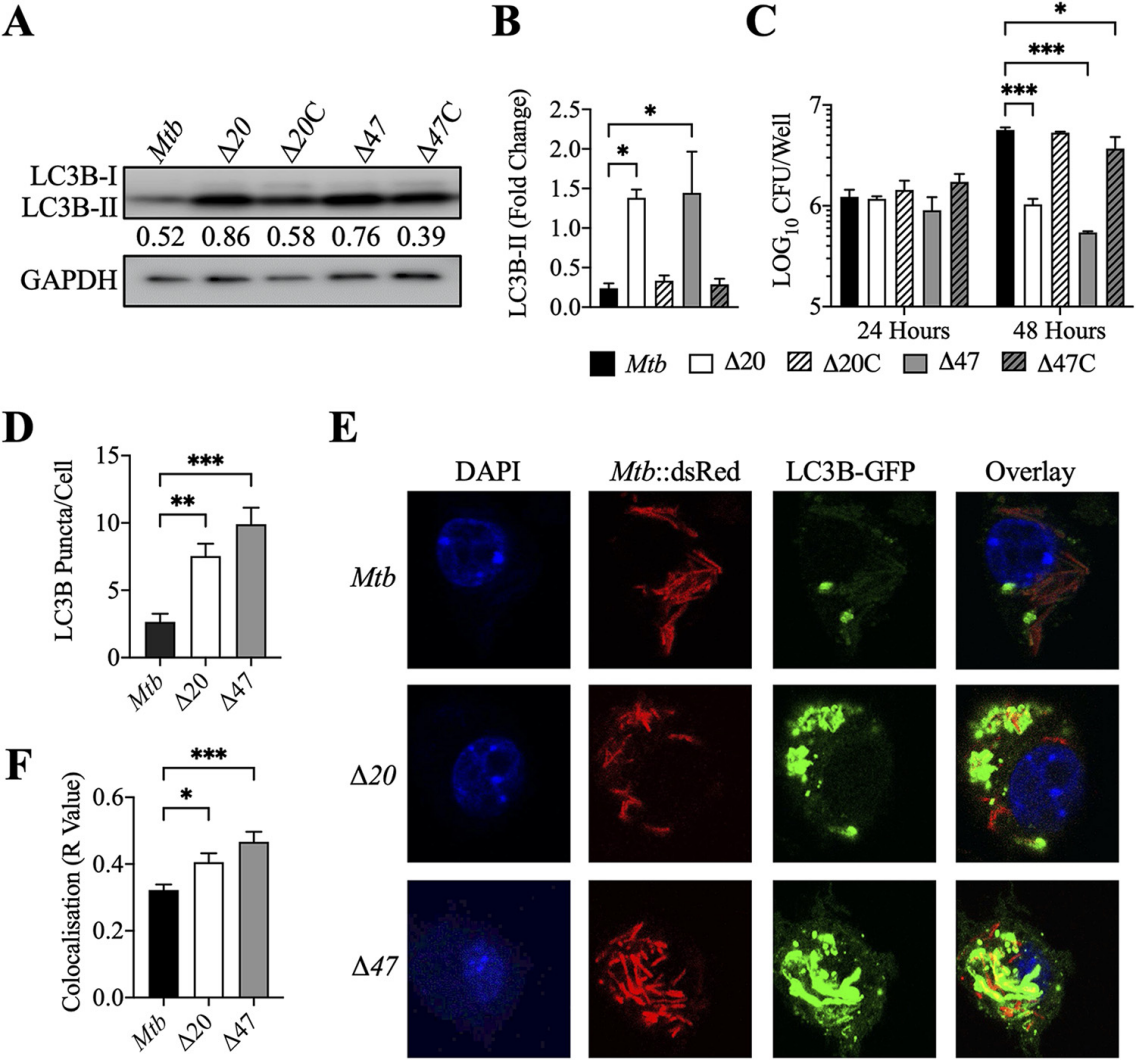
PE_PGRS20 and PE_PGRS47 of M. tuberculosis inhibit autophagy and promote bacterial survival in macrophages. (A) Immunoblots of LC3B-II accumulation in THP-1 monocyte-derived macrophages infected with M. tuberculosis, M. tuberculosis Δ*PE_PGRS* mutants, and their complemented strains. Cells were infected at an MOI of 10 and harvested 24 h postinfection. The ratio of LC3B-II compared to GAPDH is shown below the LC3B-II immunoblot. (B) Summary densitometric analysis was calculated by LC3B-II normalized to GAPDH, and then the fold change ratio was calculated compared to uninfected control for each assay. Mean ± SD of 3 independent assays shown. Significance calculated by one-way ANOVA corrected by Dunnett’s test for multiple comparisons. (C) M. tuberculosis survival was determined in THP-1 macrophages (MOI of 10) 24 and 48 h postinfection. Mean ± SD of 3 independent assays shown significance calculated by two-way ANOVA corrected by Dunnett’s test for multiple comparisons. (D) Mean ± SD of puncta per cell counted from 200 to 250 cells (from panel E). Significance is calculated by one-way ANOVA corrected by Dunnett’s test for multiple comparisons. (E) Puncta formation in LC3B-GFP RAW 264.7 macrophages was determined after infection at MOI of 10, 24 h postinfection. Green, LC3B-GFP; red, M. tuberculosis::dsRed; blue, DAPI. (F) Quantification of colocalization of LC3B-GFP puncta and dsRed bacteria was observed in panel E, and Pearson’s correlation coefficient was calculated from 200 to 250 cells. Significance calculated by one-way ANOVA corrected by Dunnett’s test for multiple comparisons. *, *P* ≤ 0.05; **, *P* ≤ 0.01; ***, *P* ≤ 0.001.

Previous studies have implicated several PE_PGRS proteins of M. tuberculosis as host cell autophagy inhibitors (6, 19). We identified *pe_pgrs20* and *pe_pgrs47* as contributors to autophagy inhibition in a loss-of-function screen of a transposon M. tuberculosis library (13). In this study, we demonstrated that both recombinant PE_PGRS proteins are secreted by Mycobacterium smegmatis. M. tuberculosis PE_PGRS20 was expressed in response to oxidative stress, a known autophagy inducer, while PE_PGRS47 was expressed in response to acidic and oxidative stress. PE_PGRS20 and PE_PGRS47 inhibited autophagy induced by M.smegmatis and reduced proinflammatory cytokine secretion during infection (18). To confirm and extend these findings, we generated strains with targeted deletions of *pe_pgrs20* (*Rv1068c*) or *pe_pgrs47* (*Rv2741*) genes in M. tuberculosis H37Rv via homologous recombination using specialized transduction (25). An increase in autophagy following infection with these mutants was observed in macrophages derived from human THP-1 cells, based on immunoblot analysis showing increased levels of LC3B-II, and this effect was reversed by genetic complementation of the deleted genes (Fig. 1A and B and Fig. S1 in the supplemental material). Inhibition of autophagy during mycobacterial infection has been previously shown to limit bacterial clearance from phagocytic cells (5). Consistent with this, both Δ*PE_PGRS20* and Δ*PE_PGRS47* strains showed reduced intracellular bacterial burdens following infection of THP-1 cells compared to infection with their complemented strains or with wild-type parental M. tuberculosis (Fig. 1C). The Δ*PE_PGRS20* and Δ*PE_PGRS47* M. tuberculosis strains also induced significantly increased autophagy in primary mouse bone marrow-derived macrophages (BMDM) compared to wild-type M. tuberculosis (Fig. S2A and B). As with THP-1 macrophages, the increased autophagy led to reduced cultivatable intracellular bacteria during Δ*PE_PGRS20* and Δ*PE_PGRS47* infection in BMDMs (Fig. S2C).

10.1128/mSphere.00549-21.1 FIG S1 Confirmation of *PE_PGRS20* and *PE_PGRS47* deletion and complementation in M. tuberculosis H37Rv. (A) Schematic representation of the predicted homologous recombination resulting in deletion of genes from M. tuberculosis H37Rv. Primers used to confirm deletions are marked by arrows (gene specific, purple; SacB specific, orange; hygromycin resistance, yellow). (B) PCR confirmation of deletions from M. tuberculosis DNA. (C) Expected PCR product sizes for PCR reactions in panel B. (D) Immunoblots showing the expression of HA-tagged PE/PPE proteins in M. tuberculosis. The expected protein sizes are as follows: PE_PGRS20, 39.3 kDa, and PE_PGRS47, 44.2 kDa. Download FIG S1, PDF file, 0.5 MB. Copyright © 2021 Strong et al. 2021 Strong et al. https://creativecommons.org/licenses/by/4.0/ This content is distributed under the terms of the Creative Commons Attribution 4.0 International license.

10.1128/mSphere.00549-21.2 FIG S2 PE_PGRS20 and PE_PGRS47 of M. tuberculosis inhibit autophagy, reducing bacterial survival in primary murine macrophages. (A) Immunoblots of LC3B-II accumulation in BMDMs infected with M. tuberculosis at MOI of 10 and harvested 24 h postinfection. B) Summary densitometric analysis was calculated by LC3B-II normalized to β-actin. Mean ± SD of 3 independent assays shown. Significance calculated by one-way ANOVA corrected by Dunnett’s test for multiple comparisons. (C) M. tuberculosis survival was determined in BMDMs (MOI of 10) at 48 h postinfection. Mean ± SD of 3 independent assays shown significance calculated by one-way ANOVA corrected by Dennett’s test for multiple comparisons. *, *P* ≤ 0.05; **, *P* ≤ 0.01; ***, *P* ≤ 0.001. Download FIG S2, PDF file, 0.6 MB. Copyright © 2021 Strong et al. 2021 Strong et al. https://creativecommons.org/licenses/by/4.0/ This content is distributed under the terms of the Creative Commons Attribution 4.0 International license.

To further assess the role of autophagy in reducing the bacterial burden in cells infected with the mutant strains, we used an autophagy reporter RAW 264.7 mouse macrophage cell line that stably expresses the LC3B-GFP fusion protein (26). These were infected with parental M. tuberculosis, Δ*PE_PGRS20*, or Δ*PE_PGRS47* bacteria constitutively expressing a dsRed fluorescent marker. At 24 h postinfection, at a multiplicity of infection (MOI) of 10, significantly more green fluorescent protein (GFP)-positive puncta were observed in cells infected with the Δ*PE_PGRS20* and Δ*PE_PGRS47* than cells infected with the wild-type parental M. tuberculosis (Fig. 1D and E). This correlated with an increased colocalization of GFP-positive puncta with red fluorescent Δ*PE_PGRS20* and Δ*PE_PGRS47* mutant bacteria (Fig. 1F).

**FIG 2:**
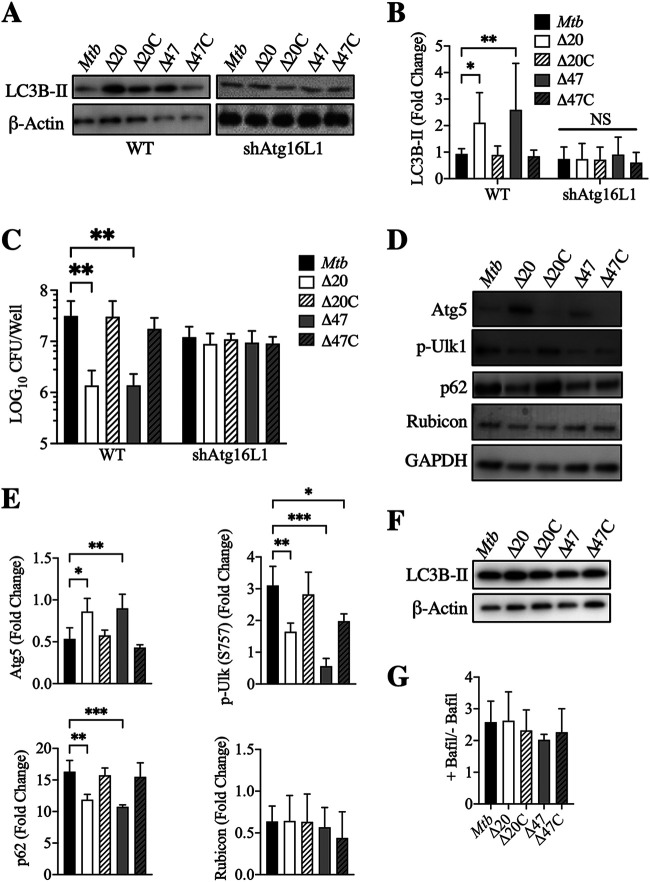
Deletion of autophagy-related genes inhibits autophagy induced by M. tuberculosis Δ*PE_PGRS* mutants. (A) Immunoblot of LC3B-II accumulation in wild-type RAW 264.7 and shAtg16L1 stably transfected macrophages infected with M. tuberculosis at MOI of 10, 24 h postinfection. (B) Densitometric summary analysis was calculated by LC3B-II normalized to β-actin, and then the fold change ratio was calculated compared to uninfected control for each assay. The mean ± SD of 3 independent assays is shown. Significance was calculated by two-way ANOVA corrected by Dunnett’s test for multiple comparisons. (C) M. tuberculosis survival was determined in wild-type RAW 264.7 and shAtg16L1 stably transfected macrophages (MOI 10) at 48 h postinfection. The mean ± SD of 3 independent assays is shown. Significance was calculated by two-way ANOVA corrected by Dunnett’s test for multiple comparisons. (D) Immunoblotting of Atg5, phospho-Ulk1 (Ser757), p62, and Rubicon in RAW 264.7 macrophages infected with M. tuberculosis at MOI of 10, 24 h postinfection. (E) Densitometric summary analysis was calculated by the protein of interest normalized to GAPDH, and then the fold change ratio was calculated compared to uninfected control for each assay. The mean ± SD of 3 independent assays is shown. Significance was calculated by one-way ANOVA corrected by Dunnett’s test for multiple comparisons. (F) Immunoblotting of LC3B-II accumulation in RAW 264.7 macrophages infected with M. tuberculosis at MOI of 10, 24 h postinfection with 10 μM bafilomycin A1 treatment. (G) Summary densiometric analysis of the ratio between LC3B-II levels from RAW 264.7 macrophages infected at MOI 10 for 24 h with and without bafilomycin A1 treatment (autophagy flux). *, *P* ≤ 0.05; **, *P* ≤ 0.01; ***, *P* ≤ 0.001.

To confirm whether these effects of *PE_PGRS20* and *PE_PGRS47* on autophagy inhibition and decreased bacterial survival were through the regulation of the canonical autophagy pathway, we analyzed the impact of genetic knockdown of autophagy-related gene 16L1 (Atg16L1), an essential component of this pathway. RAW 264.7 macrophages were stably transfected with inhibitory short hairpin RNA constructs (shAtg16L1). These transfected cells exhibited a significant reduction in Atg16L1 expression and autophagy induction during Torin-1 treatment and M. smegmatis infection (Fig. S3), both known as strong autophagy inducers via the canonical pathway (26, 27). Infection of shAtg16L1 macrophages with M. tuberculosis, Δ*PE_PGRS20* and Δ*PE_PGRS47* resulted in limited autophagy induction, suggesting that these PE_PGRS gene products inhibit canonical autophagy (Fig. 2A and B). As expected, the loss of autophagy induction resulted in the same intracellular bacterial burden in shAtg16L1 macrophages during Δ*PE_PGRS20* and Δ*PE_PGRS47* infection compared to wild-type infection at 48 h postinfection (Fig. 2C). Atg16L1 silencing had no significant effects on autophagy induction and intracellular bacterial burden during wild-type M. tuberculosis infection, indicating that M. tuberculosis actively inhibits autophagy induction.

10.1128/mSphere.00549-21.3 FIG S3 Atg16L1 knockdown in RAW 264.7 macrophages inhibits canonical autophagy. (A) Immunoblots of Atg16L1 in RAW 264.7 (WT) and RAW 264.7 shAtg16L1 macrophages. (B) Immunoblots of LC3B-II (with or without bafilomycin A1 treatment), phospho-S6 (Ser235/236), and phospho-Ulk1 (Ser757) accumulation in RAW 264.7 and RAW 264.7 shAtg16L1 macrophages 3 h posttreatment with potent autophagy induces Torin-1 (10 μM) and M. smegmatis (MOI of 10). Download FIG S3, PDF file, 1.4 MB. Copyright © 2021 Strong et al. 2021 Strong et al. https://creativecommons.org/licenses/by/4.0/ This content is distributed under the terms of the Creative Commons Attribution 4.0 International license.

To further confirm that these PE_PGRS proteins inhibit autophagy induction and not alternative autophagy-related processes such as LC3-associated phagocytosis (LAP) or autophagic flux, immunoblots for additional components of the autophagy machinery were analyzed. Infection of RAW 264.7 macrophages with Δ*PE_PGRS20* and Δ*PE_PGRS47* resulted in increased Atg5 and decreased p62 (SQSTM1) and phospho-Unc-51-like autophagy-activating kinase 1 (Ulk1) compared to wild-type M. tuberculosis infection (Fig. 2D and E). Atg5 is an essential component of the autophagosome machinery, and increased Atg5 suggests autophagy activation during Δ*PE_PGRS20* and Δ*PE_PGRS47* infection. Reduced induction of the phosphorylation of Ulk1 at the mTORC1 phosphorylation site (Ser-757) also indicated autophagy activation through inhibition of the negative autophagy regulator, mTOR. Reduced p62 (an indicator of autophagic flux) accumulation indicated autophagosomes and their cargo were being degraded by autophagy during Δ*PE_PGRS20* and Δ*PE_PGRS47* infection (Fig. 2D and E). Bafilomycin A1 inhibits autophagy flux by blocking autophagosome degradation by lysosomes. Accumulation of similar levels of LC3B-II (autophagosomes) was observed in bafilomycin-treated cells (Fig. 2F and G), once again indicating that autophagy flux was not impaired in infected macrophages. To further support the conclusion that these *pe_pgrs* gene products did not modulate LAP, rubicon expression was measured (Fig. 2D and E) since an increase in rubicon levels is associated with LAP but not with canonical autophagy (28, 29). No significant differences in rubicon levels were observed following infection with either wild-type, mutant, or complemented bacteria, which reinforced the conclusion that *pe_pgrs20* and *pe_pgrs47* inhibited canonical autophagy but not LAP.

### Inhibition of autophagy by direct expression of PE_PGRS proteins in macrophages.

**FIG 3:**
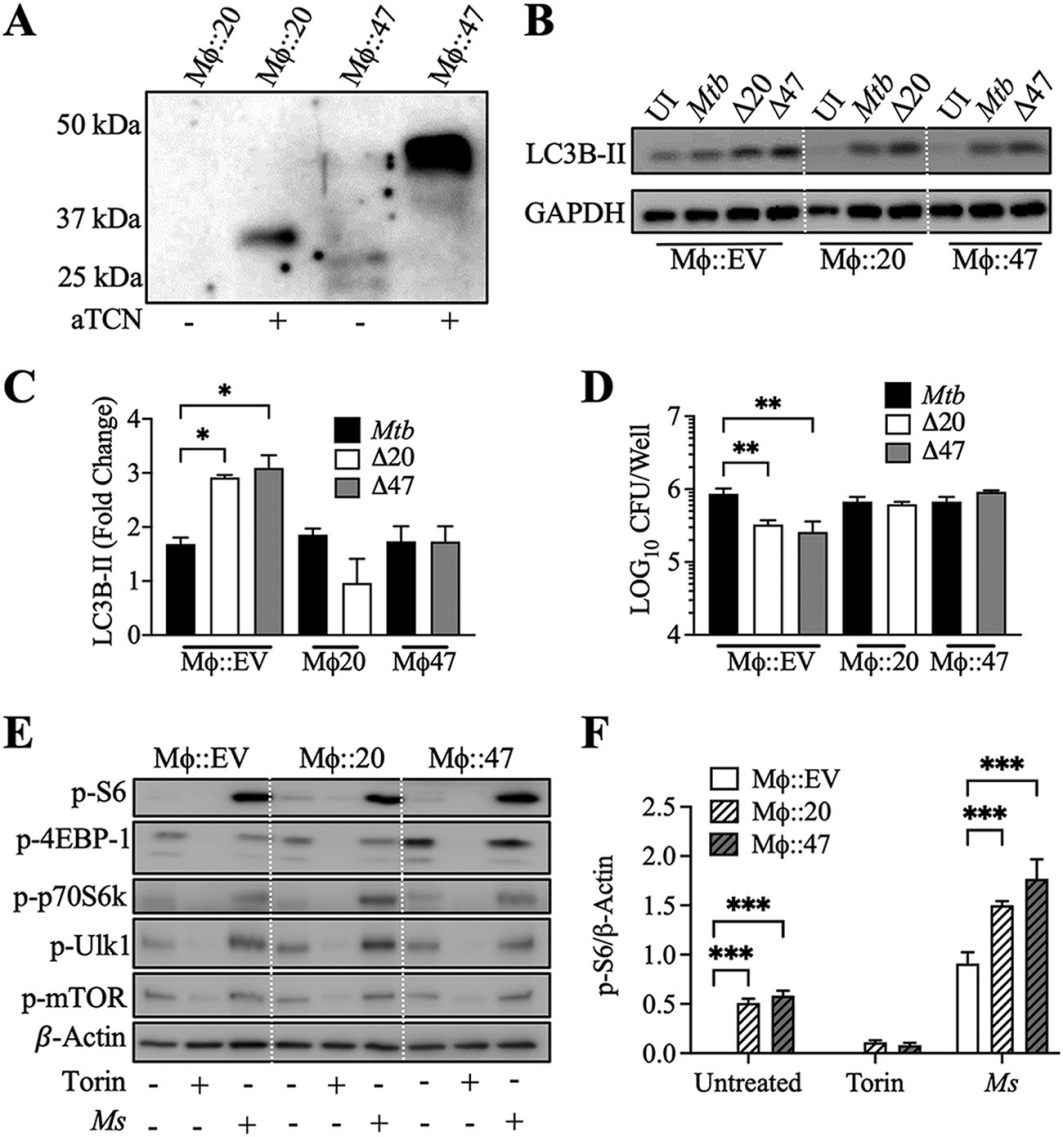
Expression of PE_PGRS proteins in RAW 264.7 macrophages inhibits autophagy during M. tuberculosis infection. (A) Anti-HA immunoblot of whole-cell lysates of RAW 264.7 macrophages stably transfected with inducible PE_PGRS expression constructs confirming expression of HA-tagged PE_PGRS20 (39.3 kDa) and PE_PGRS47 (44.2 kDa) after anhydrous tetracycline induction. (B) RAW 264.7 macrophages induced to express PE_PGRS20 and PE_PGRS47 or empty vector control were infected with M. tuberculosis and respective Δ*PE_PGRS* knockouts. Cells were lysed 24 h after infection for immunoblot assay. Immunoblots of LC3B-II accumulation in macrophages expressing PE_PGRS20 or PE_PGRS47 and infected at MOI of 10 followed by harvest at 24 h postinfection are shown. (C) Summary densitometric analysis was calculated by LC3B-II normalized to GAPDH, and then the fold change ratio was calculated compared to uninfected control for each assay. Mean ± SD of 3 independent assays is shown. Significance was calculated by two-way ANOVA corrected by Dunnett’s test for multiple comparisons. (D) Survival of M. tuberculosis and respective Δ*PE_PGRS* knockout mutants was determined in RAW 264.7 macrophages expressing PE_PGRS20 or PE_PGR47 (MOI of 10) 24 h postinfection. Mean ± SD of 3 independent assays shown, and significance was calculated by two-way ANOVA corrected by Dunnett’s test for multiple comparisons. (E) Immunoblotting of phospho-S6 (Ser235/236), phospho-4E-BP1 (Thr37/46), phospho-p70S6k (Thr389), phospho-Ulk1 (Ser757), and phospho-mTOR (Ser2448) accumulation in RAW 264.7 macrophages expressing PE_PGRS20 or PE_PGRS47 after 3 h Torin-1 or M. smegmatis (MOI of 1) treatment. (F) Summary densiometric analysis of the ratio between p-S6 and β-actin was calculated. Mean ± SD of 3 independent assays shown. Significance calculated by two-way ANOVA corrected by Dunnett’s test for multiple comparisons. *, *P* ≤ 0.05; **, *P* ≤ 0.01; ***, *P* ≤ 0.001.

To probe the mechanisms by which PE_PGRS20 and PE_PGRS47 inhibited autophagy, we generated RAW 264.7 macrophage lines with an inducible cytosolic expression of these mycobacterial proteins. RAW 264.7 macrophages were stably transduced using a lentiviral transfection system with tetracycline-inducible *pe_pgrs20* and *pe_pgrs47* genes. Both PE_PGRS20 and PE_PGRS47 were expressed at the expected full-length size following anhydrous tetracycline (aTCN) induction (Fig. 3A). Infection of RAW 264.7 macrophages expressing either of the PE_PGRS proteins with M. tuberculosis or Δ*PE_PGRS* strains demonstrated no statistical difference in LC3B-II levels; however, infection of RAW 264.7 cells expressing empty vector control with the Δ*PE_PGRS20* or Δ*PE_PGRS47* strains showed a significantly higher LC3B-II level (Fig. 3B and C) than wild-type infection. Consistent with our earlier findings, increased autophagy in macrophages infected with Δ*PE_PGRS20* and Δ*PE_PGRS47* mutants resulted in reduced intracellular bacteria 24 h postinfection; the expression of PE_PGRS20 or PE_PGRS47 in macrophages inhibited autophagy activation and subsequent intracellular mutant M. tuberculosis killing (Fig. 3D). Moreover, autophagy inhibition by PE_PGRS expression had no significant effects on autophagy induction and intracellular bacterial burden during wild-type M. tuberculosis infection, further confirming that M. tuberculosis actively inhibits autophagy induction.

We further demonstrated the direct expression of PE_PGRS20 and PE_PGRS47 to inhibit autophagy during potent autophagy induction using Torin-1 and M. smegmatis. With both of these stimuli, PE_PGRS20 or PE_PGRS47 expressing RAW 264.7 macrophages demonstrated reduced autophagy induction compared to the empty vector control RAW 264.7 macrophages. In previous studies, we demonstrated that PE/PPE proteins of M. tuberculosis, when expressed in M. smegmatis, inhibit autophagy by maintaining the activation of mTOR and downstream targets (18). Similar to these results, a significantly increased level of mTOR activation was observed, as measured by S6, 4EBP-1, p70S6 kinase, and Ulk1 phosphorylation during M. smegmatis infection of PE_PGRS expressing RAW 264.7 macrophages compared to the empty vector control macrophages (Fig. 3E and F).

**FIG 4:**
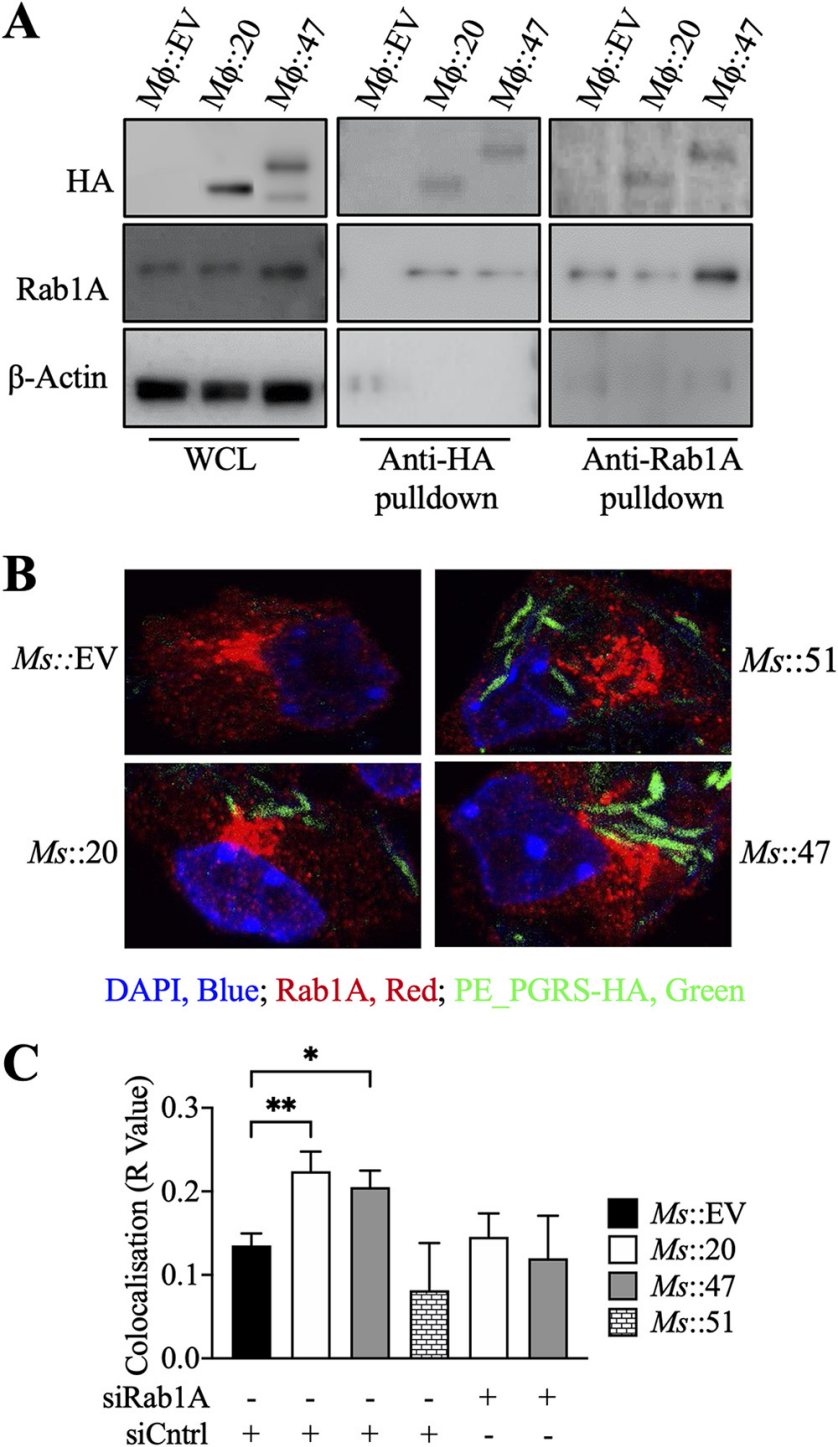
PE_PGRS20 and PE_PGRS47 interact with Rab1A in RAW 264.7 macrophages. (A) Lysates from RAW 264.7 macrophages induced to express PE_PGRS proteins were coimmunoprecipitated with anti-HA, specific for PE_PGRS HA-tagged proteins, and anti-Rab1A. (B) RAW 264.7 macrophages were transfected with scrambled siRNA (Cntrl) or Rab1A siRNA. Induced macrophages were infected with M. smegmatis::Empty Vector, M. smegmatis::PE_PGRS20, M. smegmatis::PE_PGRS47, or M. smegmatis::PPE51 at MOI of 10 for 3 h. Cells were then stained with fluorescein isothiocyanate (FITC)-conjugated anti-HA (PE/PPE specific; green) and anti-Rab1A (Alexa Fluor 555; red) to determine subcellular localization. Confocal microscopy analysis was performed to visualize the colocalization of PE_PGRS proteins and Rab1A in the context of mycobacterial infection. (C) Pearson's correlation coefficient was calculated on 200 to 250 cells from panel B. Significance was calculated by one-way ANOVA corrected by Dunnett’s test for multiple comparisons unless otherwise stated. *, *P* ≤ 0.05; **, *P* ≤ 0.01; ***, *P* ≤ 0.001.

### Interaction of PE_PGRS20 and PE_PGRS47 with Rab1A.

Using our stably transduced macrophage cell lines expressing PE_PGRS proteins, we carried out immunoprecipitation assays to identify host cell interacting partners of PE_PGRS20 and PE_PGRS47. We conducted mass spectroscopy (MS) analysis of host proteins immunoprecipitated with hemagglutinin (HA)-tagged PE_PGRS proteins. Proteins identified in cells expressing PE_PGRS proteins, but not in empty vector control by immunoprecipitations, are summarized in Table 1. As our data showed that both PE_PGRS20 and PE_PGRS47 similarly inhibit autophagy, we focused on coprecipitated host proteins identified in both PE_PGRS20 and PE_PGRS47-expressing cells. Among these, we chose to further study Ras-related protein Rab-1A (30), galectin 3 (31), and the Ras GTPase-activating-like protein (IQGAP1) (32) because of their known roles in autophagy. We excluded the nonspecific contaminant proteins found in previous immunoprecipitation studies (33). Western blot analysis of subsequent immunoprecipitations identified Galectin 3 and IQGAP in induced and uninduced samples, potentially suggesting nonspecific interactions (data not shown). However, Rab1A was confirmed by immunoprecipitation with anti-HA and anti-Rab1A as a specific interacting partner of PE_PGRS20 and PE_PGRS47 proteins (Fig. 4A; Fig. S4).

**TABLE 1:** Proteins identified from immunoprecipitation by mass spectrometry^*a*^

Protein name	GenPept accession no.	No. of peptides (no. unique) in:		Mass (kDa)
		PE_PGRS20	PE_PGRS47	
PE_PGRS20				
Protein flightless-1 homolog	Q9JJ28	7 (7)		144.7
Dedicator of cytokinesis protein 2	Q8C3J5	4 (4)		211.6
Myb-binding protein 1A	Q7TPV4	2 (2)		151.9
Trifunctional enzyme subunit alpha. Mitochondrial	Q8BMS1	2 (2)		82.6
PE_PGRS47				
Lysozyme C-1	P17897		2 (1)	16.8
PE_PGRS20 and PE_PGRS47				
Ras GTPase-activating-like protein IQGAP1	Q9JKF1	13 (13)	5 (5)	188.6
MICOS complex subunit Mic19	Q9CRB9	6 (6)	3 (3)	26.3
Endoplasmic reticulum chaperone BiP	P20029	4 (3)	10 (9)	72.4
Erythrocyte band 7 integral membrane protein	P54116	4 (4)	5 (5)	31.4
14-3-3 protein gamma	P61982	4 (3)	3 (3)	28.3
Endoplasmin	P08113	4 (4)	5 (5)	92.4
ATP synthase subunit beta, mitochondrial	P56480	3 (3)	3 (3)	56.3
Sodium/potassium-transporting ATPase subunit alpha-1	Q8VDN2	3 (3)	2 (2)	112.9
Galectin-3	P16110	2 (2)	2 (2)	27.5
Phosphate carrier protein, mitochondrial	Q8VEM8	2 (2)	2 (2)	39.6
Ras-related protein Rab-1A	P62821	2 (2)	2 (2)	22.7

a Proteins were identified in duplicate samples from two independent experiments. Proteins identified in empty vector control and nonspecific proteins commonly identified by these methods (33) were removed.

10.1128/mSphere.00549-21.4 FIG S4 PE_PGRS proteins and not PPE proteins interact with Rab1A in RAW 264.7 macrophages. Lysates from RAW 264.7 macrophages induced to express PE_PGRS proteins or PPE51 were immunoprecipitated with anti-HA, specific for PE_PGRS or PPE51 HA-tagged proteins. Download FIG S4, PDF file, 0.4 MB. Copyright © 2021 Strong et al. 2021 Strong et al. https://creativecommons.org/licenses/by/4.0/ This content is distributed under the terms of the Creative Commons Attribution 4.0 International license.

To ensure that the PE_PGRS and Rab1A interaction was maintained under infection conditions, we employed the previously characterized recombinant M. smegmatis strains expressing HA-tagged PE_PGRS20 or PE_PGRS47 or PPE51 (18). Rab1A was silenced in RAW 264.7 macrophages by transfection with Rab1A small interfering RNA (siRNA). Rab1A knockdown and control macrophages were infected with recombinant M. smegmatis at an MOI of 10 for 3 h and assayed for colocalization of PE_PGRS and Rab1A by immunofluorescence. Increased colocalization of HA-tagged PE_PGRS proteins and Rab1A was observed during M. smegmatis infection of control scrambled siRNA-transfected cells compared to M. smegmatis empty vector or PPE51-expressing controls. In contrast, Rab1A siRNA-transfected macrophages infected with the recombinant M. smegmatis or uninfected macrophages did not demonstrate increased colocalization of Rab1A and PE_PGRS proteins (Fig. 4B and C).

**FIG 5:**
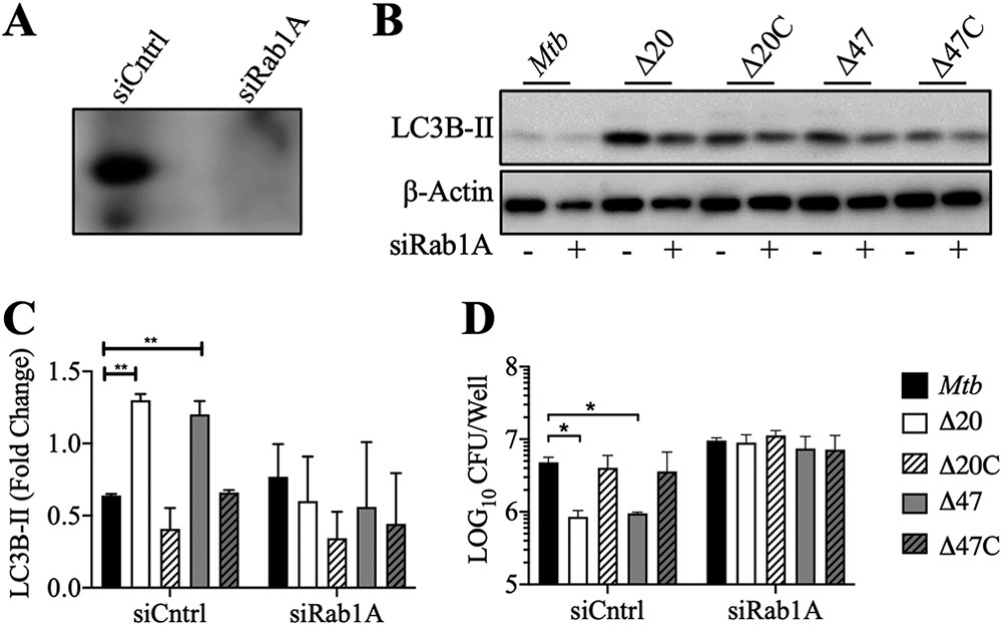
Inactivation of Rab1A inhibits autophagy during M. tuberculosis infection. (A) Immunoblotting for Rab1A of RAW 264.7 macrophages was performed after transfection with scrambled siRNA (Cntrl) or Rab1 siRNA. (B) Immunoblotting of LC3B-II accumulation was done in RAW 264.7 macrophages after Rab1A siRNA transfection infected with M. tuberculosis at MOI of 10 for 24 h. (C) Summary densitometric analysis was calculated by LC3B-II normalized to β-actin, and then the fold change ratio was calculated compared to uninfected control for each assay. Mean ± SD from 3 independent assays shown. Significance was calculated by one-way ANOVA corrected by Dunnett’s test for multiple comparisons. (D) M. tuberculosis survival was determined in Rab1A siRNA-transfected RAW 264.7 macrophages (MOI of 10) 24 h postinfection. Mean ± SD from 3 independent assays is shown. Significance was calculated by two-way ANOVA corrected by Dunnett’s test for multiple comparisons. *, *P* ≤ 0.05; **, *P* ≤ 0.01; ***, *P* ≤ 0.001.

Our data support the view that inhibition of autophagy by PE_PGRS20 and PE_PGRS47 provides M. tuberculosis a survival advantage within phagocytic cells. To determine if this survival advantage depended on Rab1A, we examined LC3B-II accumulation and bacterial survival in Rab1A-silenced macrophages (Fig. 5A). Rab1A-silenced macrophages were infected with M. tuberculosis or Δ*PE_PGRS* strains at an MOI of 10 for 24 h. No difference in LC3B-II accumulation was observed in Rab1A-silenced cells infected with wild-type M. tuberculosis or the Δ*PE_PGRS* mutants (Fig. 5B and C). At 24 h postinfection, no difference in bacterial survival was evident in Rab1A-silenced cells. In line with previous data, Δ*PE_PGRS* strains showed significantly decreased survival compared to the parental M. tuberculosis strain in scrambled siRNA control macrophages (Fig. 5D). As with Atg16L1 silencing, no significant effects on autophagy induction and intracellular bacterial burden during wild-type M. tuberculosis infection were observed in control or Rab1A-silenced cells, further showing that M. tuberculosis actively inhibits autophagy induction via Rab1A. As expected, Torin-1 and M. smegmatis did not increase autophagy when Rab1A was silenced in empty vector control RAW 264.7 macrophages (Fig. S5A). Similarly, PE_PGRS-expressing macrophages transfected with control siRNAs demonstrated less LC3B-II accumulation than empty vector-expressing macrophages when autophagy was induced (Fig. S5B).

10.1128/mSphere.00549-21.5 FIG S5 Inactivation of Rab1A inhibits autophagy similar to autophagy inhibition observed during PE_PGRS20 and PE_PGRS47 expression. (A) Immunoblotting for LC3B-II was performed from Rab1A siRNA macrophages that are induced to expresses PE_PGRS proteins. Macrophages expressing PEPGRS20 or PE_PGRS47 were treated with Torin-1 or M. smegmatis (MOI of 1) for 3 hours to induce autophagy. (B) Summary densitometric analysis was calculated by LC3B-II protein normalized to actin. Mean ± SD of 3 independent assays shown. significance calculated by two-way ANOVA corrected by Dunnett’s test for multiple comparisons. *, *P* ≤ 0.05; **, *P* ≤ 0.01; ***, *P* ≤ 0.001. Download FIG S5, PDF file, 0.1 MB. Copyright © 2021 Strong et al. 2021 Strong et al. https://creativecommons.org/licenses/by/4.0/ This content is distributed under the terms of the Creative Commons Attribution 4.0 International license.

### Inhibition of antigen presentation and cytokine secretion by PE_PGRS proteins.

**FIG 6:**
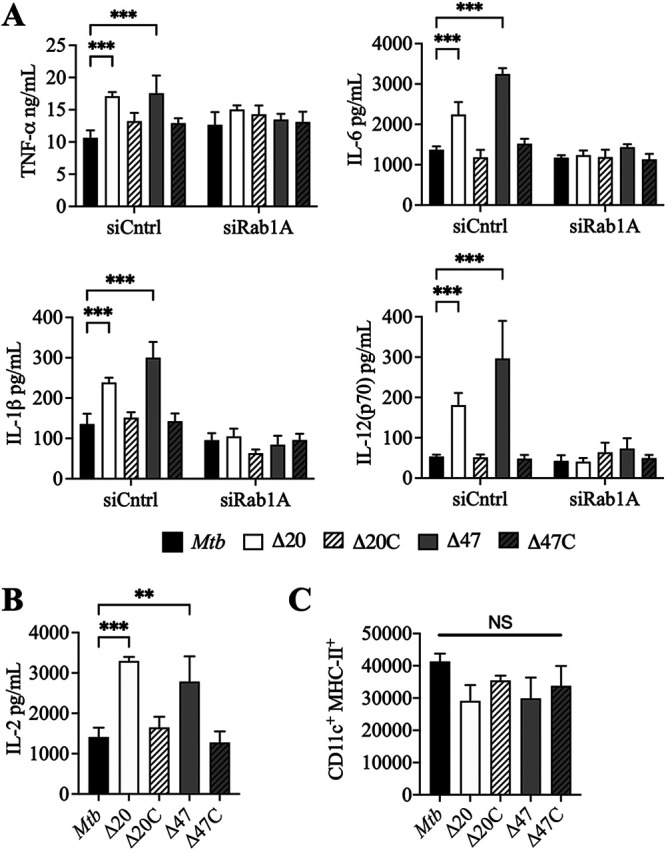
PE_PGRS20 and PE_PGRS47 inhibit proinflammatory cytokines dependent on Rab1A and antigen presentation. (A) Proinflammatory cytokine concentrations in supernatants of cultured RAW 264.7 macrophages were measured at 24 h postinfection with M. tuberculosis (MOI of 10) after transfection with scrambled siRNA (siCntrl) or Rab1A siRNA (siRab1A). Mean ± SD of experimental duplicates in 2 independent assays is shown. Significance was calculated by two-way ANOVA corrected by Dunnett’s test for multiple comparisons. (B) IL-2 concentration of culture supernatants was determined at 24 h postinfection of cocultured BMDCs and Ag85B T cell hybridomas. Significance was calculated by one-way ANOVA corrected by Dunnett’s test for multiple comparisons. Mean ± SD of 2 independent assays is shown. Significance was calculated by one-way ANOVA and corrected by Dunnett’s test for multiple comparisons. (C) BMDCs infected with M. tuberculosis for 24 h were surface stained with anti-CD11c-PE and anti-MHC class II APC and analyzed by flow cytometry. Mean fluorescence intensity (MFI) of MHC class II-APC in CD11C-positive BMDCs of 3 independent assays is shown. Significance was calculated by one-way ANOVA and corrected by Dunnett’s test for multiple comparisons. *, *P* ≤ 0.05; **, *P* ≤ 0.01; ***, *P* ≤ 0.001.

Mycobacteria are known to induce host proinflammatory cytokines that can stimulate autophagic responses. Autophagy induction modulates the transcription, processing, and secretion of many proinflammatory cytokines, resulting in a negative feedback regulation (34). The loss of bacterial autophagy inhibition has previously been shown to increase proinflammatory cytokine secretion (16, 34, 35). RAW 264.7 macrophages infected with Δ*PE_PGRS* mutants increased their secretion of interleukin 6 (IL-6), IL-1β, tumor necrosis factor alpha (TNF-α), and IL-12(p70) compared to M. tuberculosis wild-type infected cells, and this increased secretion of proinflammatory cytokines was inhibited by silencing of Rab1A (Fig. 6A).

While enhanced proinflammatory cytokine secretion has been shown to be necessary for *in vivo* control of M. tuberculosis infection, MHC class II antigen presentation also confers host advantage against the mycobacteria (36, 37). Autophagy has been identified as a route by which pathogenic antigens are processed and delivered to MHC class II molecules to present them to CD4-positive (CD4^+^) T cells (38, 39). To assess the ability of Δ*PE_PGRS20* and Δ*PE_PGRS47* to modulate MHC class II-mediated antigen presentation during infection, we infected primary murine bone marrow-derived dendritic cells (BMDCs) and cocultured infected cells with T cell hybridomas specific for the MHC-II M. tuberculosis Ag85b epitope. These hybridomas showed enhanced IL-2 secretion during infection with Δ*PE_PGRS* mutants over M. tuberculosis (Fig. 6B). As observed in previous studies with Δ*PE_PGRS47*, this established that the inhibition of autophagy by Δ*PE_PGRS20* and Δ*PE_PGRS47* leads to suppression of MHC class II antigen presentation without influencing the total surface level of MHC class II proteins of infected BMDCs (Fig. 6C).

**FIG 7:**
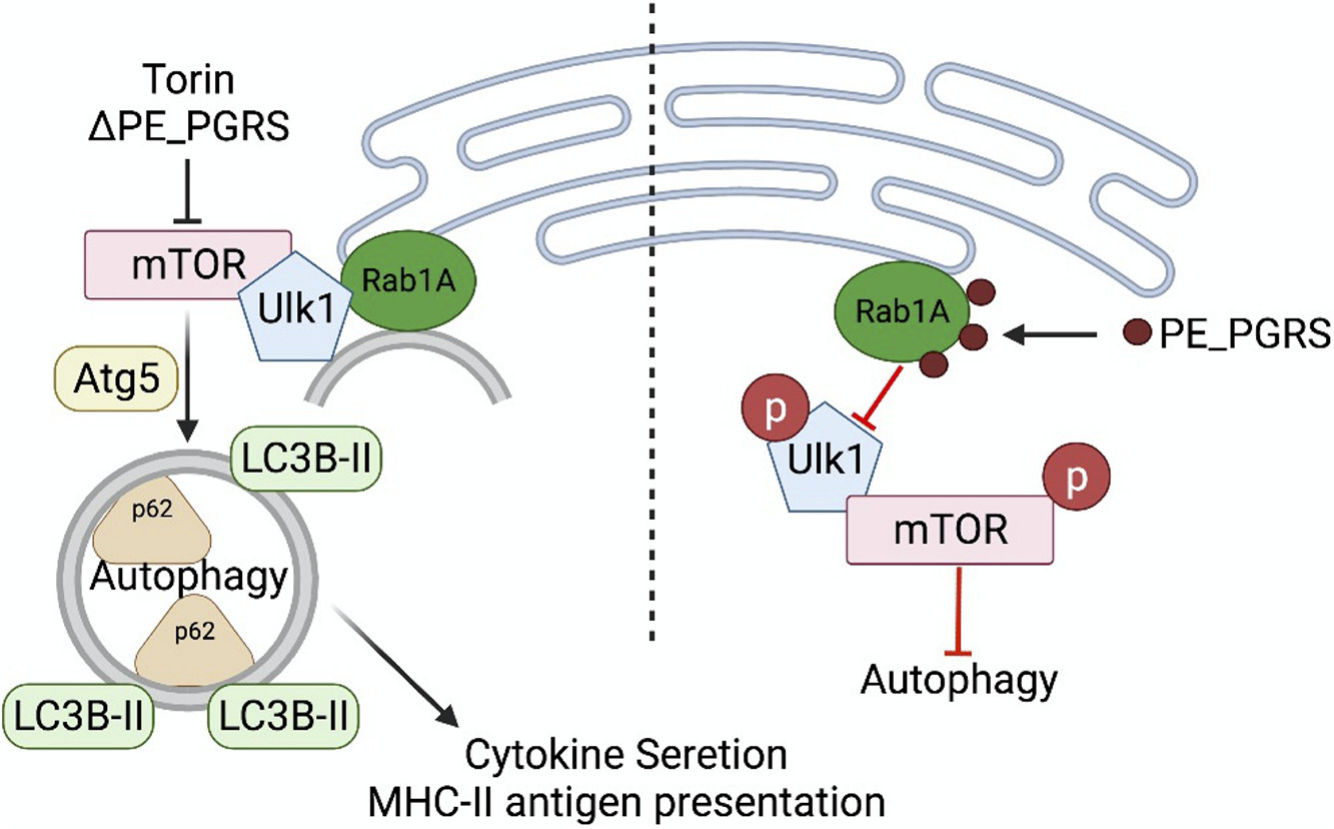
Model of PE_PGRS20 and PE_PGRS47 autophagy inhibition. Rab1A is found to be colocalized with preautophagosomes on the endoplasmic reticulum of host cells. Upon the induction of autophagy, dephosphorylated Ulk1 and its complex translocate to Rab1A and the preautophagosome. Activated Ulk1 complex recruits and activates the Beclin 1 complex, allowing the autophagosome to undergo elongation. Ubiquitinated cargo is sequestered to the elongating autophagosome by LC3B interacting regions of the sequestration machinery. Completion of autophagy results in degradation of cargo, cytokine secretion, and MHC class II-restricted antigen presentation. During M. tuberculosis infection, PE_PGRS proteins are deployed by the bacteria to bind to Rab1A. This PE_PGRS interaction with Rab1A inhibits the translocation of the Ulk1 complex to the preautophagosome and subsequent autophagy inhibition. The figure was generated using https://BioRender.com.

In total, these data support an essential role for PE_PGRS20 and PE_PGRS47 interactions with the host protein Rab1A. Based on our findings, we propose a model for autophagy inhibition by these PE_PGRS proteins by interaction with Rab1A (illustrated schematically in Fig. 7). In this model, PE_PGRS20 and PE_PGRS47 are expressed and secreted by the wild-type M. tuberculosis. These proteins directly interact with Rab1A, found on the preautophagosome (also known as the phagophore), at the endoplasmic reticulum. This interaction between mycobacterial proteins and host Rab1A prevents translocation of the Ulk1 complex to the preautophagosome, which is the first step in autophagy initiation. Inhibition of autophagy by M. tuberculosis PE_PGRS proteins resulted in secondary effects, including reduced proinflammatory cytokine secretion and reduced MHC class II-restricted antigen presentation, which combine to provide the bacteria with immune evasion and survival advantages.

## DISCUSSION

Research has revealed that autophagy is an essential host pathway for controlling intracellular bacteria. However, many pathogenic intracellular bacteria, including M. tuberculosis, have evolved various molecular mechanisms to evade and subvert this autophagy-dependent control (6, 16, 19, 26, 29, 40, 41). In the case of M. tuberculosis, a previous study identified the M. tuberculosis protein SapM as an inhibitor of autophagy flux in macrophages by interaction with Rab7 (10, 42). Another mycobacterial effector, Eis, inhibits autophagy dependent on NADPH oxidase and mitochondrial reactive oxygen species. Induction of IL-10 by M. tuberculosis Eis protein also increases autophagy potential in macrophages during infection (16, 17). Similar to inhibition of autophagy and autophagy flux, the M. tuberculosis protein CpsA inhibits LAP. Like Eis, CpsA inhibition of LAP is dependent on NADPH oxidase and reactive oxygen species (29). Thus, M. tuberculosis has multiple interactions to block or subvert the autophagy pathway, underscoring the importance of this pathogen to control host cell autophagy at multiple levels.

From a high-throughput loss-of-function screening of an M. tuberculosis transposon mutant library, we previously identified 16 additional genes that contribute to autophagy inhibition. Six autophagy-inhibiting M. tuberculosis DNA regions encoded PE/PPE family proteins (18). The expression of these M. tuberculosis PE/PPE proteins in M. smegmatis confirmed that they indeed inhibit autophagy and increase intracellular bacterial persistence or replication. These effects were correlated with increased mTOR activity and also with decreased production of TNF-α and IL-1β. One of the identified PE/PPE genes from the screen was *PE_PGRS47*. We have previously demonstrated that mice infected with the Δ*PE_PGRS47* deletion mutant showed enhanced autophagy induction, attenuated growth, and a significant increase in CD4^+^ T cell responses against MHC class II presented antigens. These findings suggested a nonredundant role for PE_PGRS47 in modulating innate intracellular host responses and the generation of adaptive immune responses that are critical to the control of M. tuberculosis (6). However, the mechanism by which PE_PGRS47 or other PE_PGRS proteins mediate autophagy-regulating function(s) remains to be determined.

This study revealed that PE_PGRS20 and PE_PGRS47 regulate host autophagy by the same molecular mechanism. Immunoprecipitation and colocalization by microscopy of macrophages stably expressing PE_PGRS20 and PE_PGRS47 demonstrated that the host protein Rab1A interacts with these PE_PGRS proteins. Rab proteins are a superfamily of small GTPases that can recruit effector proteins, forming complexes. Many studies have established that these GTPase proteins play an essential role in autophagy (30, 43, 44). Several bacterial effectors target Rab proteins to inhibit autophagy during infection. One of the host proteins frequently targeted by these bacterial effectors is Rab1A, which is essential for recruiting the Ulk1 complex to the preautophagosome to initiate autophagy (45). Intracellular pathogens such as Legionella pneumophila, Listeria monocytogenes, Salmonella enterica serovar Typhimurium, Streptococcus pyogenes, and various species of Chlamydia and Brucella all secrete bacterial effectors that interact with or inhibit Rab1A activity and thus enhance bacterial survival (41, 44, 46–50). For example, Salmonella effectors SseG and SseF bind to Rab1A, subsequently inhibiting Ulk1 activation, as evidenced by increased phosphorylation of Ulk1 and its downstream targets. Unlike SseG and SseF, SseK3 of *S.* Typhimurium modifies Rab1A by arginine GlcNAcylation (51). Interestingly, PE_PGRS20 and PE_PGRS47 share approximately 40% similarity to SseG and SseF regions of homology (52), suggesting that these proteins may have related functions.

We have previously observed that the expression of PE_PGRS proteins during mycobacterial infection of macrophages results in increased phosphorylation of mTOR (18). While the inhibition of Ulk1 recruitment by the interaction of Rab1A is established, how this mediates mTOR activation is unknown. mTORC1 impacts autophagosome biogenesis through phosphorylation of the autophagy regulatory complex formed by Ulk1 and its interacting proteins, the autophagy-related protein 13 (Atg13) and the focal adhesion kinase family interacting protein of 200 kDa (FIP200). Previous studies have demonstrated that translocation of the Ulk1 complex to the preautophagosome requires the loss of mTOR-dependent phosphorylation of Ulk1 (41, 53, 54). A possible explanation for the role of PE_PGRS proteins on mTOR regulation is provided by cancer studies. In those studies, Rab1A was shown to be a mTOR activator and an oncogene, frequently overexpressed in human cancer (55–57). Rab1A overexpression promotes mTORC1 signaling and oncogenic growth in an amino acid- and mTORC1-dependent manner. Conversely, Rab1A knockdown selectively attenuates the oncogenic growth of Rab1-overexpressing cancer cells (55, 58). Thus, we hypothesize that the interaction of PE_PGRS proteins with Rab1A activates Rab1A GTP activity, resulting in mTOR activation and autophagy inhibition. In our study, Rab1A knockdown cells (siRab1A) infected with the wild-type M. tuberculosis or two complementing strains demonstrated a reduced mTOR activation (detected by p-S6) compared to the RablA-positive cells infected with the identical mycobacteria (Fig. S6), which suggests mTOR regulation by Rab1A. However, this result needs to be further confirmed to elucidate a positive correlation between Rab1A expression levels and that of different upstream or downstream mTOR targets during M. tuberculosis infection.

10.1128/mSphere.00549-21.6 FIG S6 Inactivation of Rab1A inhibits mTOR activation. (A) Immunoblotting of p-S6 accumulation was done in RAW 264.7 macrophages after Rab1A siRNA transfection infected with M. tuberculosis at MOI of 10 for 24 h. (B) Summary densitometric analysis was calculated by p-S6 protein normalized to β-actin. Significance was calculated by two-way ANOVA corrected by Dunnett’s test for multiple comparisons. *, *P* ≤ 0.05 Download FIG S6, PDF file, 1.9 MB. Copyright © 2021 Strong et al. 2021 Strong et al. https://creativecommons.org/licenses/by/4.0/ This content is distributed under the terms of the Creative Commons Attribution 4.0 International license.

Silencing of Rab1A or Atg16L1 did not affect mycobacterial survival or autophagy induction during wild-type M. tuberculosis infection (Fig. 2 and 5), providing further evidence that M. tuberculosis actively inhibits autophagy, highlighting the importance of autophagy modulation for mycobacterial survival. However, the importance of autophagy as a cellular defense mechanism in response to M. tuberculosis infection *in vivo* (59). Despite numerous *in vitro* studies emphasizing a role for autophagy in macrophages during M. tuberculosis infection (4, 5), loss of genes essential for canonical autophagy does not correlate with susceptibility to M. tuberculosis infection *in vivo* (59). The apparent insignificance of autophagy for controlling M. tuberculosis *in vivo* is likely to reflect that M. tuberculosis encodes very efficient inhibitors of canonical autophagy. Further characterization of M. tuberculosis Δ*PE_PGRS20* and Δ*PE_PGRS47* mutants using conditional Atg gene or Rab1A knockout mice could help elucidate a role of autophagy during M. tuberculosis infection *in vivo*.

Autophagy is essential for innate defense and serves as a method to capture pathogens inside autophagosomes, with subsequent fusion with lysosomes (4–6). In this way, epitopes of pathogens and intracellular antigens can be delivered to MHC class II molecules (38, 39). Therefore, it is not surprising that autophagic pathways promote MHC class II antigen presentation and may serve to alarm the adaptive immune system against pathogens. We observed increased antigen presentation in macrophages and dendritic cells (DCs) infected with Δ*PE_PGRS20* and Δ*PE_PGRS47*, suggesting that both PE_PPE proteins restrict replication of intracellular bacteria and autophagic degradation of mycobacterial antigens for MHC class II presentation to the adaptive immune system. The Δ*PE_PGRS47* mutant has previously been demonstrated to display attenuated growth and enhanced CD4^+^ T cell responses in mouse infection models (18, 60). Inhibiting antigen presentation enables M. tuberculosis to persist in early infection relatively undetected by the host immune system. Developing treatment options based upon mycobacterial autophagy inhibition would likely lead to increased bacterial clearance as well as improved long-term adaptive immune responses. These findings improve our understanding of autophagy's role during M. tuberculosis infection and provide further information about early events in innate immunity that are important for bacterial replication and disease progression.

## MATERIALS AND METHODS

### Bacterial strains and culture conditions.

All mycobacterial strains, including M. tuberculosis (strain H37Rv), *ΔPE_PGRS* mutants (hygromycin resistant), complemented strains (kanamycin resistant), or M. smegmatis (strain mc^2^155), were cultured at 37°C with shaking in Middlebrook 7H9 supplemented with 10% oleic acid-albumin-dextrose-catalase (OADC), 0.5% glycerol, and 0.02% tyloxapol or on Middlebrook 7H10 supplemented with 10% OADC and 0.5% glycerol with or without antibiotics as per requirements (hygromycin, 50 μg/ml, or kanamycin, 25 μg/ml). Escherichia coli strains DH5α and Stbl3 were used for cloning. E. coli was grown on LB agar or broth with or without antibiotics as per requirements (kanamycin, 50 μg/ml, or ampicillin, 100 μg/ml).

Gene-specific *ΔPE_PGRS* knockouts were made by allelic exchange via specialized transduction as previously described (61). Knockouts were selected for hygromycin resistance and checked by PCR (primers cataloged in Table S1 in the supplemental material). For complementation, full-length PE/PPE genes from M. tuberculosis H37Rv were amplified by PCR and cloned in-frame into pMV261, an episomal M. tuberculosis vector with a 3′ His_6_-hemagglutinin (HA) tag (6, 62). The expression constructs were electroporated into M. smegmatis or M. tuberculosis Δ*PE_PGRS* strains and selected for kanamycin resistance. The expression of PE_PGRS proteins was confirmed by immunoblotting using an anti-HA antibody.

10.1128/mSphere.00549-21.7 TABLE S1 List of primers used in the study. Download Table S1, PDF file, 0.1 MB. Copyright © 2021 Strong et al. 2021 Strong et al. https://creativecommons.org/licenses/by/4.0/ This content is distributed under the terms of the Creative Commons Attribution 4.0 International license.

### Antibodies and other reagents.

Antibodies were purchased from Cell Signaling Technology unless indicated otherwise and are catalogued in Table S2. All reagents and media purchased from Sigma-Aldrich unless otherwise stated. Torin-1 and bafilomycin A1 were purchased from Cell Signaling Technology and dissolved in dimethyl sulfoxide (DMSO).

10.1128/mSphere.00549-21.8 TABLE S2 List of antibodies used in the study. Download Table S2, PDF file, 0.1 MB. Copyright © 2021 Strong et al. 2021 Strong et al. https://creativecommons.org/licenses/by/4.0/ This content is distributed under the terms of the Creative Commons Attribution 4.0 International license.

### Cell culture.

Mouse macrophage cell lines were maintained in supplemented Dulbecco modified Eagle medium (DMEM) (high-glucose DMEM supplemented with 1% nonessential amino acids, 10% heat-inactivated fetal bovine serum [Corning], 1% HEPES, and 50 μM β-mercaptoethanol [DMEMc]) at 37°C with 5% CO_2_. Human THP-1 monocytes were maintained in supplemented RPMI 1640 medium (bicarbonate-buffered RPMI 1640 containing glutamine supplemented with 1% nonessential amino acids, 10% heat-inactivated fetal bovine serum [Corning], 1% HEPES, 1% sodium pyruvate, and 50 μM β-mercaptoethanol [RPMIc]) at 37°C with 5% CO_2_. THP-1 cells were seeded in 12-well plates at 5 × 10^5^ cells/well 72 h before infection or treatment. Monocytes were derived to macrophages by adding 10 ng/ml phorbol myristate acetate (PMA) for 48 h. Adhered derived macrophages were washed once in RPMIc and rested overnight in RPMIc before infection.

All murine macrophage assays were conducted in 12-well plates seeded at 2.5 × 10^5^ cells/well 16 h before infection or treatment. Mycobacteria were grown to an optical density at 600 nm (OD_600_) of 0.6 to 0.8. Mycobacteria suspended in RPMIc or DMEMc were used to infect the cells at an MOI of 10 unless otherwise stated. Infection was carried out for 3 h, after which cells were washed 3 times with phosphate-buffered saline (PBS) and then treated with 50 μg/ml gentamicin in complete media for 1 h to kill extracellular bacteria. Assays were conducted in RPMIc or DMEMc with 20 μg/ml gentamicin. Cells were harvested at indicated time points in radioimmunoprecipitation assay (RIPA) buffer (150 mM NaCl, 1% NP-40 or Triton X-100, 0.5% sodium deoxycholate, 0.1% SDS, 50 mM Tris-HCl, pH 8.0, and 20 mM Tris-HCl, pH 7.5) for plating for intracellular survival or analysis of autophagy induction by immunoblotting. For CFU enumeration, lysates were serially diluted and plated on 7H10 with appropriate antibiotics. Macrophages treated with Torin-1 were washed three times with PBS and then incubated in DMEMc with 10 μM Torin-1 for 3 h. Macrophages treated with M. smegmatis were washed three times with PBS and incubated with media containing M. smegmatis at indicated MOI for 3 to 6 h.

### Murine primary cell isolation.

Bone marrow-derived primary cells were derived according to previously published methods (63). Briefly, marrow was flushed from tibias and femurs of 6- to 8-week-old C57BL/6J mice aseptically and cultured in non-tissue culture-treated serological plates in RPMIc supplemented with 100 U/ml penicillin and 100 μg/ml streptomycin (RPMIcAbx). For macrophage differentiation, cells were seeded in 100-mm plates at 2 × 10^5^ cells/ml and differentiated by the addition of 15% L929 fibroblast-conditioned media for 6 days, followed by feeding with fresh media every 2 days. On day 6, adherent cells were washed with ice-cold PBS and detached by incubation on ice for 20 to 30 min in ice-cold PBS. For dendritic cell differentiation, cells were seeded in 100-mm plates at 2 × 10^5^ cells/ml and differentiated by the addition of 20 ng/ml recombinant granulocyte-macrophage colony-stimulating factor (rGM-CSF) (BioLegend), with feeding every 3 days. On day 10, adherent cells were washed with ice-cold PBS and detached by incubation with ice-cold PBS containing 5 mM EDTA for 5 min.

### Macrophage transductions.

RAW 264.7 macrophages were stably transduced with the second-generation lentiviral Tet-on vector pInducer20 (64) in which full-length PE_PGRS genes from M. tuberculosis had been cloned using gateway cloning. Atg16L1 short hairpin RNA (shRNA) constructs were purchased from Horizon Inspired Cell Solution, constructed by the RNAi Consortium (TRC-Mm1.0 library; clone IDs TRCN0000173438, TRCN0000175121, TRCN0000175371, TRCN0000175562, and TRCN0000176385).

RAW 264.7 macrophages were transduced using recombinant lentivirus. Lentivirus was generated using ViromerRed (OriGene Technologies, MD) according to the manufacturer’s instructions in the human embryonic kidney (HEK) 293T cells. Briefly, expression constructs (pInducer or shRNA, 1.64 pmol), packaging (psPAX2, 1.3 pmol), and envelope (pMD2.G, 0.72 pmol) plasmids were added to ViromerRed and then added to cells. HEK293T cell culture supernatant was collected at 48 h posttransfection and polybrene added at 10 μg/ml. Recombinant lentivirus was added to RAW 264.7 macrophages and incubated for 24 h, after which media containing lentivirus was removed and fresh DMEMc added. At 48 h postaddition of lentivirus, macrophages were treated with 10 μg/ml puromycin or 400 μg/ml G418. Single puromycin- or G418-resistant cells were expanded and assayed for Atg16L1 expression or PE_PGRS expression after induction with 500 ng/ml anhydrous tetracycline (aTcn) for 16 h.

### Rab1A knockdown.

Rab1A was silenced in RAW 264.7 macrophages by transfection with Rab1A siRNA (Thermo Fisher Scientific). Rab1A siRNA or scrambled siRNA (Cell Signaling Technology) at a concentration of 30 nM was added to Lipofectamine 2000 (Invitrogen) as per the manufacturer’s instructions and incubated for 10 min at room temperature. Lipidated siRNA was diluted 1:10 with Opti-MEM (Gibco) and added to RAW 264.7 macrophages. Before adding siRNA, RAW 264.7 macrophages grown in DMEMc were transferred to Opti-MEM media supplemented with 5% fetal bovine serum (FBS) and incubated for 3 h. Macrophages were incubated for 48 h and then seeded for infection as described above. The efficiency of Rab1A knockdown was assayed by immunoblot for each transfection.

### Immunoblotting.

Cellular protein was prepared in 1× radioimmunoprecipitation assay (RIPA) buffer, and the protein concentration was determined by bicinchoninic acid (BCA) assay (Pierce). Aliquots of lysates containing 1 to 10 μg of protein were resolved on 12% SDS-PAGE gels at 180 V for 40 min. Proteins were transferred to 0.2 μm polyvinylidene difluoride (PVDF) using a Bio-Rad TransBlot Turbo at 2.5 amps and 25 V for 5 to 10 min depending on molecular weight. PVDF membranes were blocked in 5% nonfat dry milk in 1× Tris-buffered saline (TBS) plus 0.1% Tween 20 (TBST) or OneBlock Western-CL blocking buffer (Genesee Scientific) for LC3B blots at room temperature for at least 1 h. Primary antibodies at 1:5,000 dilution were incubated overnight at 4°C in TBST. Anti-rabbit IgG-horseradish peroxidase (HRP) antibody (1:10,000) was added to membranes for 45 min in TBST. Proteins of interest were revealed using Clarity ECL (Bio-Rad) according to the manufacturer’s instructions. Films were scanned, and densitometric analysis was conducted by ImageJ software (https://imagej.nih.gov). The protein of interest was normalized to β-actin or GAPDH (glyceraldehyde-3-phosphate dehydrogenase) loading control to calculate autophagy levels (6).

### Confocal microscopy imaging.

Macrophage assays were carried out as described above, with macrophages allowed to adhere to glass coverslips. At 24 h postinfection, macrophages were washed with PBS and fixed in 4% paraformaldehyde for 48 h before mounting coverslips. For antibody staining assays, macrophage assays were carried out as described above, with macrophages allowed to adhere to glass coverslips. Coverslips were fixed in 4% paraformaldehyde for 15 min and then permeabilized using 0.02% saponin in PBS with 5% bovine serum albumin (BSA) for 20 min. Coverslips were then washed and blocked in PBS with 5% BSA, 0.01% saponin, and 0.2% Tween 20 for 20 min. Macrophage-coated coverslips were incubated with anti-Rab1A (1:100) in blocking solution overnight at 4°C. Coverslips were washed three times in blocking solution and incubated for 1 h in Alexa Fluor 555-conjugated anti-rabbit secondary antibody (1:1,000) and Alexa Fluor 488-conjugated anti-HA (1:500). Coverslips were then mounted in ProLong Gold with DAPI (4′,6-diamidino-2-phenylindole; Cell Signaling Technology) at 4°C overnight. Images were taken on a Zeiss laser scanning microscope 880 and analyzed with ImageJ software.

### Immunoprecipitation.

RAW 264.7 macrophages induced to express PE_PGRS proteins were lysed in lysis buffer (20 mM Tris-HCl [pH 7.5], 150 mM NaCl, 0.2% Nonidet P-40, and cOmplete protease inhibitor [Roche]) and centrifuged at 12,000 × *g* for 20 min. The supernatant was cleared using rabbit serum conjugated to magnetic beads for 16 h at 4°C. Cleared lysates were incubated with magnetic beads conjugated to anti-HA or anti-Rab1A antibodies for 5 h at 4°C. Beads were washed 3 times using lysis buffer. Bound proteins were analyzed by Western blotting.

### ELISA.

At indicated time points, cell culture supernatants were collected for cytokine analysis. BioLegend enzyme-linked immunosorbent assay (ELISA) Max Deluxe kits were used as per the manufacturer’s instructions. Briefly, plates were coated in capture antibody for 16 h and blocked in blocking buffer for 1 h at room temperature. A total of 100 μl of standards and culture supernatant was added and incubated for 2 h at room temperature. The detection antibody was incubated for 1 h, followed by avidin-HRP for 30 min. Next, TMB (3,3′,5,5′-tetramethylbenzidine) substrate was incubated for 20 min, and sulfuric acid was added to stop the reaction. Absorbance was read at 450 nm, and background absorbance (570 nm) was subtracted.

### Antigen presentation assay.

Dendritic cells were seeded in 96-well plates at 5 × 10^5^ cells/ml and allowed to adhere overnight. Cells were infected as per macrophage infection as described above. After washing infected cells 3 times with PBS, T cell hybridomas specific for the I-Ab Ag85B epitope (33) were seeded at 5 × 10^5^ cells/ml on top of the infected dendritic cells and incubated for a further 21 h. Culture supernatants were collected, and assays for IL-2 secretion were performed by ELISA.

### Statistics.

GraphPad Prism 8 was used for all analyses. Analysis of variance (ANOVA) was used to determine significance with Dunnett correction for multiple comparisons. A *P* value of <0.05 was considered to be significant.

## References

[1] Chai Q, Zhang Y, Liu CH. 2018. Mycobacterium tuberculosis: an adaptable pathogen associated with multiple human diseases. Front Cell Infect Microbiol 8:158. doi:10.3389/fcimb.2018.00158. 29868514 PMC5962710

[2] World Health Organization. 2021. Global tuberculosis report 2020. World Health Organization, Geneva, Switzerland.

[3] Awuh JA, Flo TH. 2017. Molecular basis of mycobacterial survival in macrophages. Cell Mol Life Sci 74:1625–1648. doi:10.1007/s00018-016-2422-8. 27866220 PMC11107535

[4] Gutierrez MG, Master SS, Singh SB, Taylor GA, Colombo MI, Deretic V. 2004. Autophagy is a defense mechanism inhibiting BCG and Mycobacterium tuberculosis survival in infected macrophages. Cell 119:753–766. doi:10.1016/j.cell.2004.11.038. 15607973

[5] Castillo EF, Dekonenko A, Arko-Mensah J, Mandell MA, Dupont N, Jiang S, Delgado-Vargas M, Timmins GS, Bhattacharya D, Yang H, Hutt J, Lyons CR, Dobos KM, Deretic V. 2012. Autophagy protects against active tuberculosis by suppressing bacterial burden and inflammation. Proc Natl Acad Sci USA 109:E3168–E3176. doi:10.1073/pnas.1210500109. 23093667 PMC3503152

[6] Saini NK, Baena A, Ng TW, Venkataswamy MM, Kennedy SC, Kunnath-Velayudhan S, Carreño LJ, Xu J, Chan J, Larsen MH, Jacobs WR, Porcelli SA. 2016. Suppression of autophagy and antigen presentation by Mycobacterium tuberculosis PE_PGRS47. Nat Microbiol 1:16133. doi:10.1038/nmicrobiol.2016.133. 27562263 PMC5662936

[7] Alonso S, Pethe K, Russell DG, Purdy GE. 2007. Lysosomal killing of Mycobacterium mediated by ubiquitin-derived peptides is enhanced by autophagy. Proc Natl Acad Sci USA 104:6031–6036. doi:10.1073/pnas.0700036104. 17389386 PMC1851611

[8] Zullo AJ, Jurcic Smith KL, Lee S. 2014. Mammalian target of rapamycin inhibition and mycobacterial survival are uncoupled in murine macrophages. BMC Biochem 15:4. doi:10.1186/1471-2091-15-4. 24528777 PMC3937017

[9] Jagannath C, Lindsey DR, Dhandayuthapani S, Xu Y, Hunter RL, Eissa NT. 2009. Autophagy enhances the efficacy of BCG vaccine by increasing peptide presentation in mouse dendritic cells. Nat Med 15:267–276. doi:10.1038/nm.1928. 19252503

[10] Chandra P, Ghanwat S, Matta SK, Yadav SS, Mehta M, Siddiqui Z, Singh A, Kumar D. 2015. Mycobacterium tuberculosis inhibits RAB7 recruitment to selectively modulate autophagy flux in macrophages. Sci Rep 5:16320. doi:10.1038/srep16320. 26541268 PMC4635374

[11] Watson RO, Manzanillo PS, Cox JS. 2012. Extracellular M. tuberculosis DNA targets bacteria for autophagy by activating the host DNA-sensing pathway. Cell 150:803–815. doi:10.1016/j.cell.2012.06.040. 22901810 PMC3708656

[12] Watson RO, Bell SL, MacDuff DA, Kimmey JM, Diner EJ, Olivas J, Vance RE, Stallings CL, Virgin HW, Cox JS. 2015. The cytosolic sensor cGAS detects Mycobacterium tuberculosis DNA to induce type I interferons and activate autophagy. Cell Host Microbe 17:811–819. doi:10.1016/j.chom.2015.05.004. 26048136 PMC4466081

[13] Wild P, Farhan H, McEwan DG, Wagner S, Rogov V.v, Brady NR, Richter B, Korac J, Waidmann O, Choudhary C, Dötsch V, Bumann D, Dikic I. 2011. Phosphorylation of the autophagy receptor optineurin restricts Salmonella growth. Science 333:228–233. doi:10.1126/science.1205405. 21617041 PMC3714538

[14] Zheng YT, Shahnazari S, Brech A, Lamark T, Johansen T, Brumell JH. 2009. The adaptor protein p62/SQSTM1 targets invading bacteria to the autophagy pathway. J Immunol 183:5909–5916. doi:10.4049/jimmunol.0900441. 19812211

[15] Thurston TLM, Ryzhakov G, Bloor S, von Muhlinen N, Randow F. 2009. The TBK1 adaptor and autophagy receptor NDP52 restricts the proliferation of ubiquitin-coated bacteria. Nat Immunol 10:1215–1221. doi:10.1038/ni.1800. 19820708

[16] Shin D-M, Jeon B-Y, Lee H-M, Jin HS, Yuk J-M, Song C-H, Lee S-H, Lee Z-W, Cho S-N, Kim J-M, Friedman RL, Jo E-K. 2010. Mycobacterium tuberculosis Eis regulates autophagy, inflammation, and cell death through redox-dependent signaling. PLoS Pathog 6:e1001230. doi:10.1371/journal.ppat.1001230. 21187903 PMC3002989

[17] Duan L, Yi M, Chen J, Li S, Chen W. 2016. Mycobacterium tuberculosis EIS gene inhibits macrophage autophagy through up-regulation of IL-10 by increasing the acetylation of histone H3. Biochem Biophys Res Commun 473:1229–1234. doi:10.1016/j.bbrc.2016.04.045. 27079235

[18] Strong EJ, Jurcic Smith KL, Saini NK, Ng TW, Porcelli SA, Lee S. 1975. Identification of autophagy-inhibiting factors of Mycobacterium tuberculosis by high-throughput loss-of-function screening. Biochem Pharmacol 24:e00269-20. doi:10.1128/IAI.00269-20. PMC7671894 32989037

[19] Deng W, Long Q, Zeng J, Li P, Yang W, Chen X, Xie J. 2017. Mycobacterium tuberculosis PE_PGRS41 enhances the intracellular survival of M. smegmatis within macrophages via blocking innate immunity and inhibition of host defense. Sci Rep 7:46716. doi:10.1038/srep46716. 28440335 PMC5404228

[20] Cole ST, Brosch R, Parkhill J, Garnier T, Churcher C, Harris D, Gordon S.v, Eiglmeier K, Gas S, Barry CE, Tekaia F, Badcock K, Basham D, Brown D, Chillingworth T, Connor R, Davies R, Devlin K, Feltwell T, Gentles S, Hamlin N, Holroyd S, Hornsby T, Jagels K, Krogh A, McLean J, Moule S, Murphy L, Oliver K, Osborne J, Quail MA, Rajandream M-A, Rogers J, Rutter S, Seeger K, Skelton J, Squares R, Squares S, Sulston JE, Taylor K, Whitehead S, Barrell BG. 1998. Deciphering the biology of Mycobacterium tuberculosis from the complete genome sequence. Nature 393:537–544. doi:10.1038/31159. 9634230

[21] Singh VK, Berry L, Bernut A, Singh S, Carrère-Kremer S, Viljoen A, Alibaud L, Majlessi L, Brosch R, Chaturvedi V, Geurtsen J, Drancourt M, Kremer L. 2016. A unique PE_PGRS protein inhibiting host cell cytosolic defenses and sustaining full virulence of Mycobacterium marinum in multiple hosts. Cell Microbiol 18:1489–1507. doi:10.1111/cmi.12606. 27120981

[22] Abdallah AM, Gey van Pittius NC, DiGiuseppe Champion PA, Cox J, Luirink J, Vandenbroucke-Grauls CMJE, Appelmelk BJ, Bitter W. 2007. Type VII secretion — mycobacteria show the way. Nat Rev Microbiol 5:883–891. doi:10.1038/nrmicro1773. 17922044

[23] Bottai D, Brosch R. 2009. Mycobacterial PE, PPE and ESX clusters: novel insights into the secretion of these most unusual protein families. Mol Microbiol 73:325–328. doi:10.1111/j.1365-2958.2009.06784.x. 19602151

[24] Bottai D, di Luca M, Majlessi L, Frigui W, Simeone R, Sayes F, Bitter W, Brennan MJ, Leclerc C, Batoni G, Campa M, Brosch R, Esin S. 2012. Disruption of the ESX-5 system of Mycobacterium tuberculosis causes loss of PPE protein secretion, reduction of cell wall integrity and strong attenuation. Mol Microbiol 83:1195–1209. doi:10.1111/j.1365-2958.2012.08001.x. 22340629

[25] Lee S, Kriakov J, Vilcheze C, Dai Z, Hatfull GF, Jacobs WR. 2004. Bxz1, a new generalized transducing phage for mycobacteria. FEMS Microbiol Lett 241:271–276. doi:10.1016/j.femsle.2004.10.032. 15598543

[26] Zullo AJ, Lee S. 2012. Mycobacterial induction of autophagy varies by species and occurs independently of mammalian target of rapamycin inhibition. J Biol Chem 287:12668–12678. doi:10.1074/jbc.M111.320135. 22275355 PMC3339952

[27] Leontieva O, Blagosklonny MV. 2016. Gerosuppression by pan-mTOR inhibitors. Aging (Albany NY) 8:3535–3551. doi:10.18632/aging.101155. 28077803 PMC5270685

[28] Martinez J, Malireddi RKS, Lu Q, Cunha LD, Pelletier S, Gingras S, Orchard R, Guan J-L, Tan H, Peng J, Kanneganti T-D, Virgin HW, Green DR. 2015. Molecular characterization of LC3-associated phagocytosis reveals distinct roles for Rubicon, NOX2 and autophagy proteins. Nat Cell Biol 17:893–906. doi:10.1038/ncb3192. 26098576 PMC4612372

[29] Köster S, Upadhyay S, Chandra P, Papavinasasundaram K, Yang G, Hassan A, Grigsby SJ, Mittal E, Park HS, Jones V, Hsu F-F, Jackson M, Sassetti CM, Philips JA. 2017. Mycobacterium tuberculosis is protected from NADPH oxidase and LC3-associated phagocytosis by the LCP protein CpsA. Proc Natl Acad Sci USA 114:E8711–E8720. doi:10.1073/pnas.1707792114. 28973896 PMC5642705

[30] Ao X, Zou L, Wu Y. 2014. Regulation of autophagy by the Rab GTPase network. Cell Death Differ 21:348–358. doi:10.1038/cdd.2013.187. 24440914 PMC3921601

[31] Chauhan S, Kumar S, Jain A, Ponpuak M, Mudd MH, Kimura T, Choi SW, Peters R, Mandell M, Bruun J-A, Johansen T, Deretic V. 2016. TRIMs and galectins globally cooperate and TRIM16 and galectin-3 co-direct autophagy in endomembrane damage homeostasis. Dev Cell 39:13–27. doi:10.1016/j.devcel.2016.08.003. 27693506 PMC5104201

[32] Lu R, Herrera BB, Eshleman HD, Fu Y, Bloom A, Li Z, Sacks DB, Goldberg MB. 2015. Shigella effector OspB activates mTORC1 in a manner that depends on IQGAP1 and promotes cell proliferation. PLoS Pathog 11:e1005200. doi:10.1371/journal.ppat.1005200. 26473364 PMC4608727

[33] Trinkle-Mulcahy L, Boulon S, Lam YW, Urcia R, Boisvert F-M, Vandermoere F, Morrice NA, Swift S, Rothbauer U, Leonhardt H, Lamond A. 2008. Identifying specific protein interaction partners using quantitative mass spectrometry and bead proteomes. J Cell Biol 183:223–239. doi:10.1083/jcb.200805092. 18936248 PMC2568020

[34] Harris J. 2011. Autophagy and cytokines. Cytokine 56:140–144. doi:10.1016/j.cyto.2011.08.022. 21889357

[35] Songane M, Kleinnijenhuis J, Netea MG, van Crevel R. 2012. The role of autophagy in host defence against Mycobacterium tuberculosis infection. Tuberculosis (Edinb) 92:388–396. doi:10.1016/j.tube.2012.05.004. 22683183

[36] Mogues T, Goodrich ME, Ryan L, LaCourse R, North RJ. 2001. The relative importance of T cell subsets in immunity and immunopathology of airborne Mycobacterium tuberculosis infection in mice. J Exp Med 193:271–280. doi:10.1084/jem.193.3.271. 11157048 PMC2195922

[37] Baena A, Porcelli SA. 2009. Evasion and subversion of antigen presentation by Mycobacterium tuberculosis. Tissue Antigens 74:189–204. doi:10.1111/j.1399-0039.2009.01301.x. 19563525 PMC2753606

[38] Dengjel J, Schoor O, Fischer R, Reich M, Kraus M, Müller M, Kreymborg K, Altenberend F, Brandenburg J, Kalbacher H, Brock R, Driessen C, Rammensee H-G, Stevanovic S. 2005. Autophagy promotes MHC class II presentation of peptides from intracellular source proteins. Proc Natl Acad Sci USA 102:7922–7927. doi:10.1073/pnas.0501190102. 15894616 PMC1142372

[39] Paludan C, Schmid D, Landthaler M, Vockerodt M, Kube D, Tuschl T, Münz C. 2005. Endogenous MHC class II processing of a viral nuclear antigen after autophagy. Science 307:593–596. doi:10.1126/science.1104904. 15591165

[40] Xu J, Laine O, Masciocchi M, Manoranjan J, Smith J, Du SJ, Edwards N, Zhu X, Fenselau C, Gao L-Y. 2007. A unique Mycobacterium ESX-1 protein co-secretes with CFP-10/ESAT-6 and is necessary for inhibiting phagosome maturation. Mol Microbiol 66:787–800. doi:10.1111/j.1365-2958.2007.05959.x. 17908204

[41] Feng Z-Z, Jiang A-J, Mao A-W, Feng Y, Wang W, Li J, Zhang X, Xing K, Peng X. 2018. The Salmonella effectors SseF and SseG inhibit Rab1A-mediated autophagy to facilitate intracellular bacterial survival and replication. J Biol Chem 293:9662–9673. doi:10.1074/jbc.M117.811737. 29610274 PMC6016468

[42] Hu D, Wu J, Wang W, Mu M, Zhao R, Xu X, Chen Z, Xiao J, Hu F, Yang Y, Zhang R. 2015. Autophagy regulation revealed by SapM-induced block of autophagosome-lysosome fusion via binding RAB7. Biochem Biophys Res Commun 461:401–407. doi:10.1016/j.bbrc.2015.04.051. 25896765

[43] Kern A, Dikic I, Behl C. 2015. The integration of autophagy and cellular trafficking pathways via RAB GAPs. Autophagy 11:2393–2397. doi:10.1080/15548627.2015.1110668. 26565612 PMC4835203

[44] Huang J, Birmingham CL, Shahnazari S, Shiu J, Zheng YT, Smith AC, Campellone KG, do Heo W, Gruenheid S, Meyer T, Welch MD, Ktistakis NT, Kim PK, Klionsky DJ, Brumell JH. 2011. Antibacterial autophagy occurs at PI(3)P-enriched domains of the endoplasmic reticulum and requires Rab1 GTPase. Autophagy 7:17–26. doi:10.4161/auto.7.1.13840. 20980813 PMC3039730

[45] Wang J, Menon S, Yamasaki A, Chou H-T, Walz T, Jiang Y, Ferro-Novick S. 2013. Ypt1 recruits the Atg1 kinase to the preautophagosomal structure. Proc Natl Acad Sci USA 110:9800–9805. doi:10.1073/pnas.1302337110. 23716696 PMC3683756

[46] Mukherjee S, Liu X, Arasaki K, McDonough J, Galán JE, Roy CR. 2011. Modulation of Rab GTPase function by a protein phosphocholine transferase. Nature 477:103–106. doi:10.1038/nature10335. 21822290 PMC3206611

[47] Cortes C, Rzomp KA, Tvinnereim A, Scidmore MA, Wizel B. 2007. Chlamydia pneumoniae inclusion membrane protein Cpn0585 interacts with multiple Rab GTPases. Infect Immun 75:5586–5596. doi:10.1128/IAI.01020-07. 17908815 PMC2168330

[48] Dong N, Zhu Y, Lu Q, Hu L, Zheng Y, Shao F. 2012. Structurally distinct bacterial TBC-like GAPs link Arf GTPase to Rab1 inactivation to counteract host defenses. Cell 150:1029–1041. doi:10.1016/j.cell.2012.06.050. 22939626

[49] Toh H, Nozawa T, Minowa-Nozawa A, Hikichi M, Nakajima S, Aikawa C, Nakagawa I. 2020. Group A Streptococcus modulates RAB1- and PIK3C3 complex-dependent autophagy. Autophagy 16:334–346. doi:10.1080/15548627.2019.1628539. 31177902 PMC6984453

[50] Uchiya K, Tobe T, Komatsu K, Suzuki T, Watarai M, Fukuda I, Yoshikawa M, Sasakawa C. 1995. Identification of a novel virulence gene, virA, on the large plasmid of Shigella, involved in invasion and intercellular spreading. Mol Microbiol 17:241–250. doi:10.1111/j.1365-2958.1995.mmi_17020241.x. 7494473

[51] Meng K, Zhuang X, Peng T, Hu S, Yang J, Wang Z, Fu J, Xue J, Pan X, Lv J, Liu X, Shao F, Li S. 2020. Arginine GlcNAcylation of Rab small GTPases by the pathogen Salmonella Typhimurium. Commun Biol 3:287. doi:10.1038/s42003-020-1005-2. 32504010 PMC7275070

[52] Madeira F, Park YM, Lee J, Buso N, Gur T, Madhusoodanan N, Basutkar P, Tivey ARN, Potter SC, Finn RD, Lopez R. 2019. The EMBL-EBI search and sequence analysis tools APIs in 2019. Nucleic Acids Res 47:W636–W641. doi:10.1093/nar/gkz268. 30976793 PMC6602479

[53] Itakura E, Mizushima N. 2010. Characterization of autophagosome formation site by a hierarchical analysis of mammalian Atg proteins. Autophagy 6:764–776. doi:10.4161/auto.6.6.12709. 20639694 PMC3321844

[54] Mizushima N. 2010. The role of the Atg1/ULK1 complex in autophagy regulation. Curr Opin Cell Biol 22:132–139. doi:10.1016/j.ceb.2009.12.004. 20056399

[55] Cheng Z, Shao X, Xu M, Wang J, Kuai X, Zhang L, Wu J, Zhou C, Mao J. 2019. Rab1A promotes proliferation and migration abilities via regulation of the HER2/AKT-independent mTOR/S6K1 pathway in colorectal cancer. Oncol Rep 41:2717–2728. doi:10.3892/or.2019.7071. 30896866 PMC6448090

[56] Wang X, Liu F, Qin X, Huang T, Huang B, Zhang Y, Jiang B. 2016. Expression of Rab1A is upregulated in human lung cancer and associated with tumor size and T stage. Aging (Albany NY) 8:2790–2798. doi:10.18632/aging.101087. 27902464 PMC5191870

[57] Thomas JD, Zhang Y-J, Wei Y-H, Cho J-H, Morris LE, Wang H-Y, Zheng XFS. 2014. Rab1A is an mTORC1 activator and a colorectal oncogene. Cancer Cell 26:754–769. doi:10.1016/j.ccell.2014.09.008. 25446900 PMC4288827

[58] Xu M, Shao X, Kuai X, Zhang L, Zhou C, Cheng Z. 2019. Expression analysis and implication of Rab1A in gastrointestinal relevant tumor. Sci Rep 9:13384. doi:10.1038/s41598-019-49786-7. 31527621 PMC6746845

[59] Kimmey JM, Huynh JP, Weiss LA, Park S, Kambal A, Debnath J, Virgin HW, Stallings CL. 2015. Unique role for ATG5 in neutrophil-mediated immunopathology during M. tuberculosis infection. Nature 528:565–569. doi:10.1038/nature16451. 26649827 PMC4842313

[60] Bardarov S, Bardarov S, Pavelka MS, Sambandamurthy V, Larsen M, Tufariello J, Chan J, Hatfull G, Jacobs WR. 2002. Specialized transduction: an efficient method for generating marked and unmarked targeted gene disruptions in Mycobacterium tuberculosis, M. bovis BCG and M. smegmatis. Microbiology 148:3007–3017. doi:10.1099/00221287-148-10-3007. 12368434

[61] Stover CK, de la Cruz VF, Fuerst TR, Burlein JE, Benson LA, Bennett LT, Bansal GP, Young JF, Lee MH, Hatfull GF, Snapper SB, Barletta RG, Jacobs WR, Bloom BR. 1991. New use of BCG for recombinant vaccines. Nature 351:456–460. doi:10.1038/351456a0. 1904554

[62] Trouplin V, Boucherit N, Gorvel L, Conti F, Mottola G, Ghigo E. 2013. Bone marrow-derived macrophage production. J Vis Exp :e50966. doi:10.3791/50966. 24300014 PMC3991821

[63] Meerbrey KL, Hu G, Kessler JD, Roarty K, Li MZ, Fang JE, Herschkowitz JI, Burrows AE, Ciccia A, Sun T, Schmitt EM, Bernardi RJ, Fu X, Bland CS, Cooper TA, Schiff R, Rosen JM, Westbrook TF, Elledge SJ. 2011. The pINDUCER lentiviral toolkit for inducible RNA interference in vitro and in vivo. Proc Natl Acad Sci USA 108:3665–3670. doi:10.1073/pnas.1019736108. 21307310 PMC3048138

[64] Klionsky DJ, Abeliovich H, Agostinis P, Agrawal DK, Aliev G, Askew DS, Baba M, Baehrecke EH, Bahr BA, Ballabio A, Bamber BA, Bassham DC, Bergamini E, Bi X, Biard-Piechaczyk M, Blum JS, Bredesen DE, Brodsky JL, Brumell JH, Brunk UT, Bursch W, Camougrand N, Cebollero E, Cecconi F, Chen Y, Chin L-S, Choi A, Chu CT, Chung J, Clark RSB, Clarke PGH, Clarke SG, Clave C, Cleveland JL, Codogno P, Colombo MI, Coto-Montes A, Cregg JM, Cuervo AM, Debnath J, Dennis PB, Dennis PA, Demarchi F, Deretic V, Devenish RJ, di Sano F, Dice JF, Distelhorst CW, Dinesh-Kumar SP, Eissa NT, DiFiglia M, Djavaheri-Mergny M, Dorsey FC, Dröge W, Dron M, et al. 2008. Guidelines for the use and interpretation of assays for monitoring autophagy in higher eukaryotes. Autophagy 4:151–175. doi:10.4161/auto.5338. 18188003 PMC2654259

