# Supplementary figures and images for: Mycobacterium tuberculosis PE_PGRS20 and PE_PGRS47 Proteins Inhibit Autophagy by Interaction with Rab1A
Source: mSphere. 2021 Aug 4;6(4):e00549-21. doi: 10.1128/mSphere.00549-21 (PMC8386380; doi:10.1128/mSphere.00549-21)

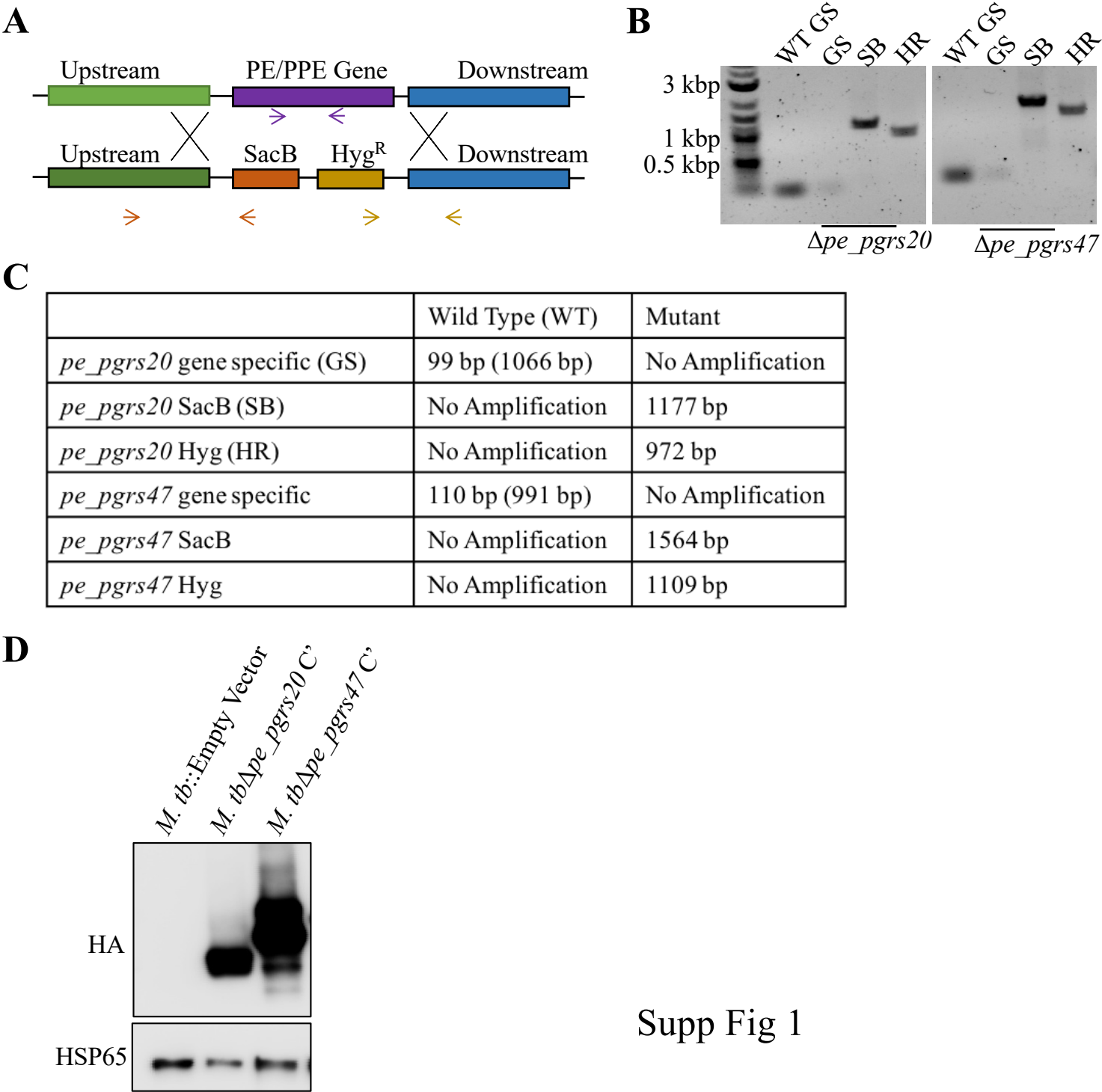

Supp Fig 1

Supplement: FIG S1 [file msphere.00549-21-sf001.pdf]

**A**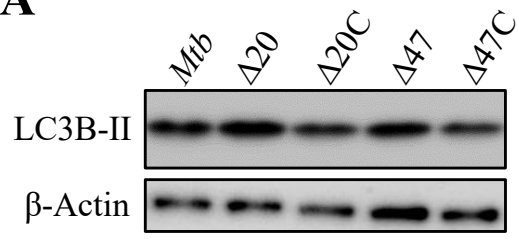**B**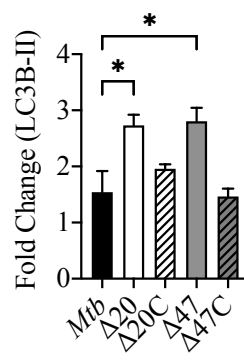**C**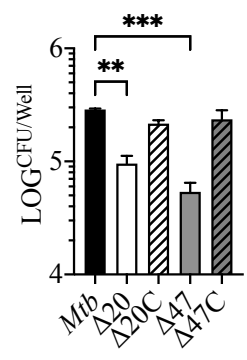

Supp Fig 2

Supplement: FIG S2 [file msphere.00549-21-sf002.pdf]

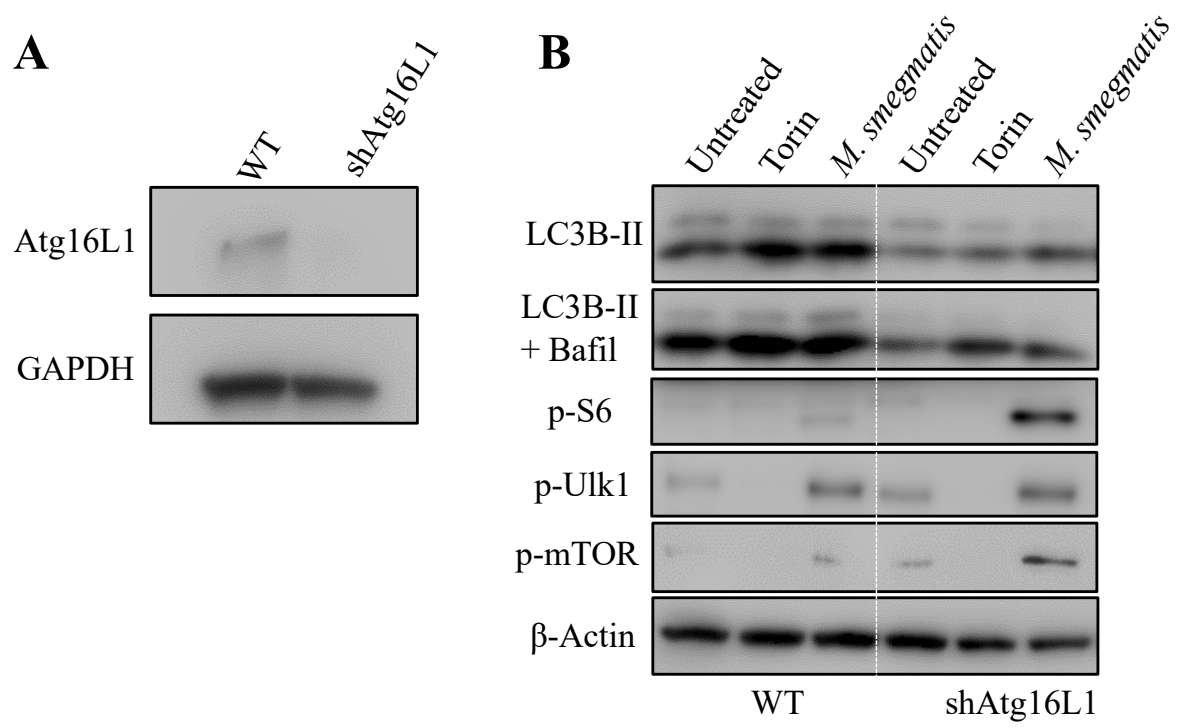

Supp Fig 3

Supplement: FIG S3 [file msphere.00549-21-sf003.pdf]

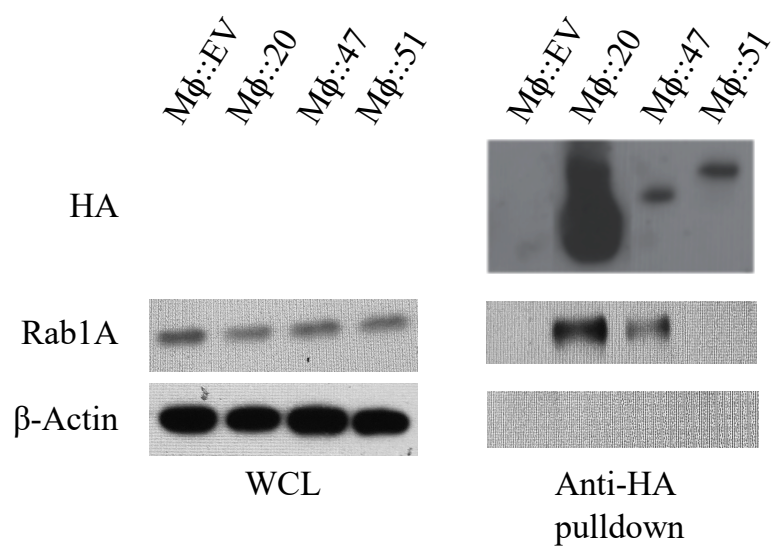

Supp Fig 4

Supplement: FIG S4 [file msphere.00549-21-sf004.pdf]

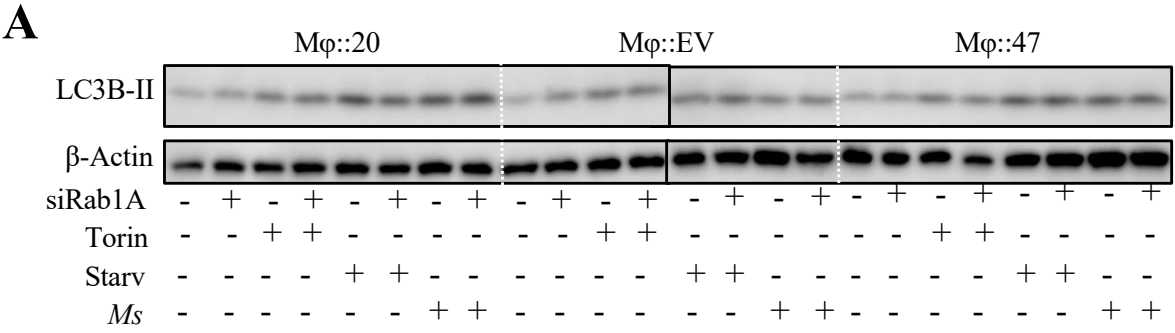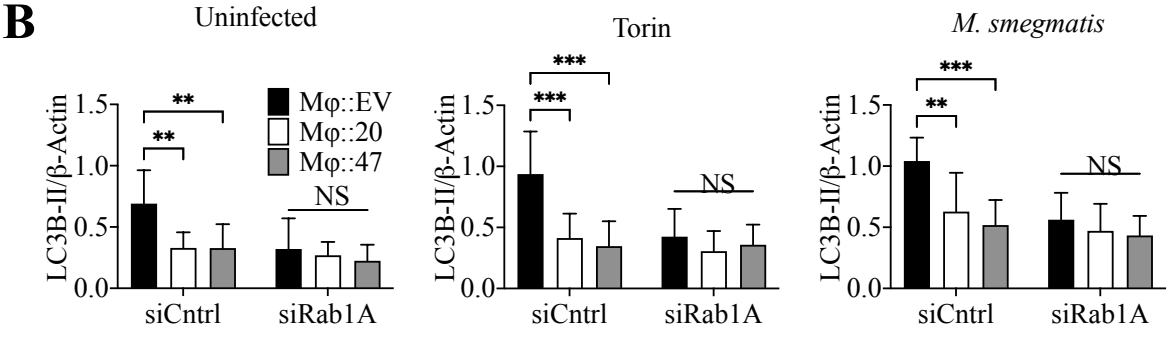

Supp Fig 5

Supplement: FIG S5 [file msphere.00549-21-sf005.pdf]

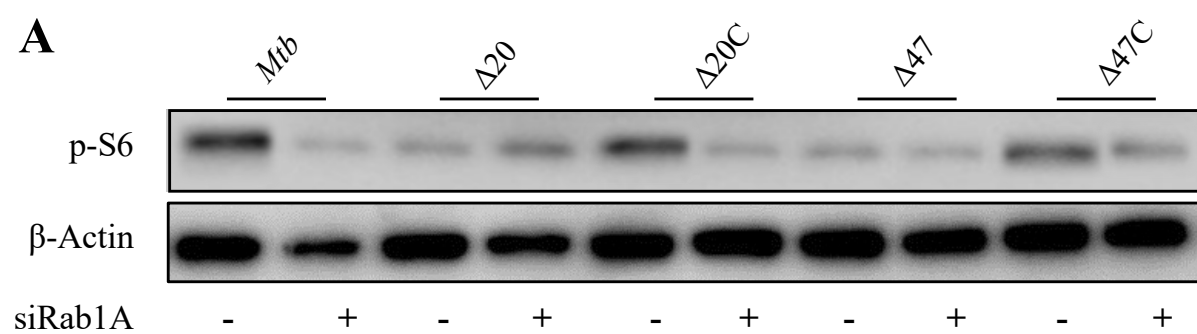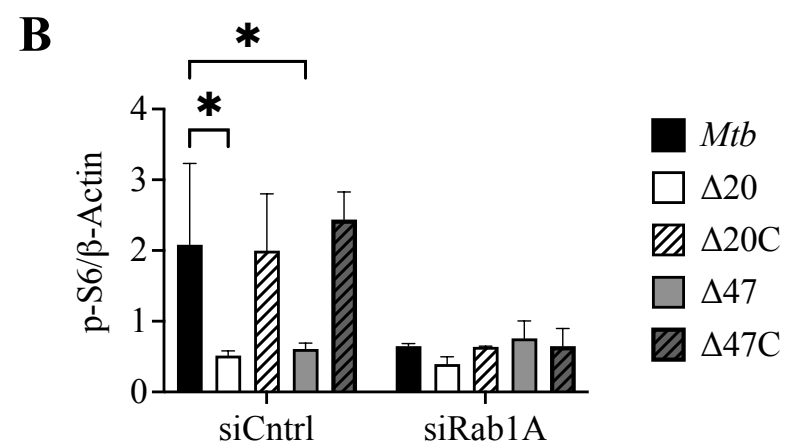

Supp Fig 6

Supplement: FIG S6 [file msphere.00549-21-sf006.pdf]
