# Supplementary material for: Mycobacterium tuberculosis PE_PGRS20 and PE_PGRS47 Proteins Inhibit Autophagy by Interaction with Rab1A
Source: mSphere. 2021 Aug 4;6(4):e00549-21. doi: 10.1128/mSphere.00549-21 (PMC8386380; doi:10.1128/mSphere.00549-21)
Supplement: TABLE S1 [file msphere.00549-21-st001.pdf]

Supplementary Table 1

Gene	Forward (3' – 5')	Reverse (3' – 5')
pe_pgrs20 Cloning	ATGGATCCAATGTCCTACATGAT	GCAAGCTTTTTGCCCGGGCGTGC
pe_pgrs47 Cloning	CGGATCCGATGTCATTTGTGATC	ATAAGCTTTGCTAGGCAGCAATCC
SacB	CGGCAGGTATATGTGATGGG	
Hyg	AACTGCTCGCCTTCACCTTC	
pe_pgrs20 Upstream	CTTCCCCAGACACGCTGGAT	
pe_pgrs47 Upstream	GTTGAGCACGGCCATCTG	
pe_pgrs20 Downstream		CAATGCCTGCACGAACTGCT
pe_pgrs47 Downstream		GCCCGTAGCGTTCGATATAC
pe_pgrs20	GATGGCAGCGTTTCATGACC	GTTACCGATCAACTGGGCGT
pe_pgrs47	CGCTTCAGCAACTAGTCGAT	TAGAACGTGCCGGCGTTACC
