# Supplementary material for: Mycobacterium tuberculosis PE_PGRS20 and PE_PGRS47 Proteins Inhibit Autophagy by Interaction with Rab1A
Source: mSphere. 2021 Aug 4;6(4):e00549-21. doi: 10.1128/mSphere.00549-21 (PMC8386380; doi:10.1128/mSphere.00549-21)
Supplement: TABLE S2 [file msphere.00549-21-st002.pdf]

Supplementary Table 2

**Antibody**	**Company**	**Catalog Number**
LC3B (D11)	Cell Signalling Technology	3868
β-Actin	Cell Signalling Technology	4970
GAPDH	Cell Signalling Technology	5174
HA-HRP	Sigma-Aldrich	12013819001
p-S6 (Ser235/236)	Cell Signalling Technology	4857
Atg16L1	Cell Signalling Technology	8089
HA-tag	Sigma-Aldrich	H3663-200UL
Atg5	Cell Signalling Technology	12994
p-Ulk1 (Ser757)	Cell Signalling Technology	14202
Rab1A	Cell Signalling Technology	13075
Rabbit IgG-Alexa Fluor555	Cell Signalling Technology	4413
HA-Tag Alexa Fluor 488 conjugate	Cell Signalling Technology	2350
Rabbit IgG-HRP	Cell Signalling Technology	7076
SQSTM1/p62	Cell Signalling Technology	5114
Rubicon	Cell Signalling Technology	8465
p-4E-BP1 (Thr37/46)	Cell Signalling Technology	2855
p-p70 S6 Kinase (Thr389)	Cell Signalling Technology	9234
p-mTOR (Ser2448)	Cell Signalling Technology	2971
CD11c PE conjugate	Cell Signalling Technology	117307
I-A^b APC conjugate	Cell Signalling Technology	116417
